# Nuclear and cytoplasmic USP30-AS1 coordinately regulate breast cancer progression through HnRNPF/p21 and EZH2/c-Myc/p21 axes

**DOI:** 10.1016/j.gendis.2025.101684

**Published:** 2025-05-10

**Authors:** Yapei Jiang, Weijie Liao, Qilei Xin, Ruonan Wang, Guanglan Lin, Jia Li, Zijian Yang, Shiyue Yang, Haowei Zhang, Xiaolin Li, Qian Peng, Yaou Zhang, Weidong Xie, Naihan Xu

**Affiliations:** a State Key Laboratory of Chemical Oncogenomics, Institute of Biopharmaceutical and Health Engineering, Tsinghua Shenzhen International Graduate School, Tsinghua University, Shenzhen, Guangdong 518055, China; b Department of Hematology and Oncology, International Cancer Center, Shenzhen University General Hospital, Shenzhen University, Shenzhen, Guangdong 518000, China; c Department of Breast and Thyroid Surgery, Peking University Shenzhen Hospital, Shenzhen, Guangdong 518034, China; d School of Food and Drug, Shenzhen Polytechnic University, Shenzhen, Guangdong 518055, China

**Keywords:** Breast cancer, c-Myc, CDKN1A/p21, HnRNPF, USP30-AS1

## Abstract

Emerging evidence suggests that aberrant expression of long non-coding RNAs (lncRNAs) is strongly associated with the occurrence and progression of breast cancer. Herein, we identified ubiquitin specific peptidase 30 antisense RNA 1 (USP30-AS1) as a markedly upregulated lncRNA in breast cancer tissues, and the transcription factor SPI1 functions upstream to regulate the expression of USP30-AS1. Gene set enrichment analysis suggests that USP30-AS1 may regulate cell proliferation. Knockdown of USP30-AS1 suppresses breast cancer cell proliferation and tumor growth by up-regulating CDKN1A/p21. Mechanistically, USP30-AS1 exhibits dual localization within breast cancer cells. In the cytoplasm, it interacts with HnRNPF, disrupting its binding to the p21 3′UTR, which destabilizes p21 mRNA and ultimately reduces p21 expression. In the nucleus, USP30-AS1 suppresses p21 transcription by enhancing the activity of c-Myc, a known transcriptional repressor of p21. USP30-AS1 binds to enhancer of zeste homolog 2 (EZH2), a histone methyltransferase, and prevents EZH2 from binding to the c-Myc promoter. This promotes epigenetic up-regulation of c-Myc by reducing H3K27 trimethylation. Together, these findings demonstrate the critical role of USP30-AS1 in breast cancer progression through HnRNPF/p21 and EZH2/c-Myc/p21 axes, highlighting its potential as a therapeutic target for breast cancer treatment.

## Introduction

Long non-coding RNAs (lncRNAs) are transcribed by RNA polymerase II, generally consisting of more than 200 nucleotides and structurally resembling mRNA molecules.^1^ LncRNAs are widely distributed in human organisms and play crucial roles in regulating gene expression at the epigenetic, transcriptional, and post-transcriptional levels. LncRNAs can interact with RNA, DNA, or proteins to modulate various cellular processes, including chromatin remodeling, transcriptional activation and repression, RNA processing and stability as well as protein translation and localization.^2^, ^3^, ^4^ LncRNAs are present in both cytoplasm and nucleus, and their subcellular localization is crucial for determining their functional roles. Nuclear lncRNAs regulate transcriptional processes by interacting with chromatin and facilitating its remodeling, whereas cytoplasmic lncRNAs interact with proteins or miRNAs, thereby modulating mRNA stability, translation, and post-translational modifications.^5^^,^^6^

Genome-wide association studies have discovered many lncRNAs linked to various human cancers.^7^ Accumulating evidence suggests that dysregulated lncRNA expression contributes to unlimited cell proliferation, disrupted differentiation, resistance to apoptosis, enhanced migration and invasion, and abnormal angiogenesis in cancer cells.^8^^,^^9^ Consequently, lncRNAs are emerging as promising candidates for diagnostic and prognostic biomarkers as well as therapeutic targets in cancer. Breast cancer, the leading cause of cancer-related deaths among women globally, is known for its high metastatic potential and poor prognosis.^10^ Increasing evidence indicates that lncRNAs play a role in tumor suppression or oncogenesis by regulating key cellular processes in breast cancer, including cell proliferation, apoptosis, migration, invasion, epithelial–mesenchymal transition, stemness, and drug resistance.^11^^,^^12^ For instance, lncRNA BC069792 is found to be down-regulated in breast cancer tissues. Overexpression of lncRNA BC069792 suppresses breast cancer cell proliferation by functioning as a molecular sponge for miR-658 and miR-4739. This interaction leads to the up-regulation of potassium voltage-gated channel subfamily Q member 4 (KCNQ4) and the inactivation of the Janus Kinase 2 (JAK2)/p-AKT signaling pathway.^13^ Elevated levels of LINC02273 have been observed in breast cancer, where it regulates metastasis and proliferation through the LINC02273-HnRNPL-AGR2 axis.^14^ Lnc-THOR is significantly up-regulated in breast cancer tissues, where it interacts with HnRNPD to stabilize PDK1 mRNA. This interaction activates the MAPK/PI3K-AKT signaling pathway, thereby promoting breast cancer progression.^15^

Ubiquitin specific peptidase 30 antisense RNA 1 (USP30-AS1) is a recently identified lncRNA located on chromosome 12 (q24.11). It is transcribed from the antisense strand of USP30, a mitochondrial-localized deubiquitinase known to inhibit mitophagy.^16^^,^^17^ USP30-AS1 is up-regulated in cervical cancer, glioblastoma, and acute myeloid leukemia, where it plays a role in promoting tumor development. In cervical cancer cells, depletion of USP30-AS1 suppresses cell proliferation and invasion while promoting apoptosis through the USP30-AS1/miR-299-3p/PTP4A1 axis.^18^ USP30-AS1 inhibits mitophagy and disrupts mitochondrial homeostasis in glioblastoma cells, a mechanism associated with poorer survival outcomes in both primary and recurrent glioma patients.^19^ In acute myeloid leukemia, USP30-AS1 promotes disease progression through the cis-regulation of adjacent genes.^20^ Conversely, USP30-AS1 is down-regulated in colon cancer tissues, and its enforced expression suppresses malignant progression by targeting miR-765, suggesting a tumor-suppressive role.^21^ These findings highlight USP30-AS1's dual regulatory role in cancer development. However, its specific involvement in breast cancer pathogenesis remains unclear and requires further investigation.

In this investigation, we found that USP30-AS1 is significantly up-regulated in breast cancer and is closely associated with signaling pathways that regulate cell proliferation. Knockdown of USP30-AS1 reduced breast cancer cell proliferation and tumor growth by modulating the expression of CDKN1A/p21, c-Myc, and cyclin D1. Conversely, the addition of exogenous USP30-AS1 had the opposite effect. Mechanistically, cytoplasmic USP30-AS1 binds to HnRNPF, thereby preventing its interaction with p21 mRNA and leading to p21 mRNA destabilization. Additionally, nuclear USP30-AS1 interacts with enhancer of zeste homolog 2 (EZH2) and epigenetically regulates c-Myc expression. Our data demonstrated that USP30-AS1 plays an oncogenic function in breast cancer through HnRNPF/p21 and EZH2/c-Myc/p21 axes.

## Materials and methods

### Bioinformatic analyses

RNA sequencing (RNA-seq) data were obtained from The Cancer Genome Atlas (TCGA), Gene Expression Omnibus (GEO), and Cancer Cell Line Encyclopedia (CCLE) databases. Since USP30-AS1 is an antisense transcript of USP30, meaning that its transcription direction is opposite to that of USP30, and base calling can distinguish between the two by analyzing the transcript direction in RNA-seq data: the transcript of USP30 is read in one direction, while that of USP30-AS1 is read in the opposite direction. By aligning sequencing data to the reference transcriptome, base calling can further identify specific exon-intron structures or splice variants, thereby precisely distinguishing between the transcripts of the two genes. Using the alignment tool HISAT2 and annotation tool StringTie in combination with base calling results can provide detailed transcript annotation information, enabling accurate differentiation between USP30 and USP30-AS1 transcripts. Expression data of USP30-AS1 were further extracted and the median value in different groups was further analyzed using the Mann–Whitney U test.

### Cell culture

MDA-MB-231, MCF-7, and HEK-293 cell lines were obtained from American Type Culture Collection (ATCC, Manassas, USA). MDA-MB-231 and HEK-293 cells were cultured in DMEM medium with 4.5 g/L d-glucose (Gibco, New York State, USA), MCF-7 cells were cultured in RPMI 1640 Medium (Gibco, New York State, USA). All mediums were supplemented with 10% FBS (Biowest, France) and 1% Antibiotic-Antimycotic (Sangon Biotech, #E607011). Cells were incubated in a 37 °C humid incubator containing 5% CO_2_.

### Transfection and construction of stable cell lines

The siRNAs targeting USP30-AS1, HnRNPF and negative control (siNC) and the respective shRNAs were synthesized by Gene Pharma (Shanghai, China). The sequences are listed in Table S1 and Table S2. The pcDNA3.1-USP30-AS1 and pcDNA3.1-HnRNPF were constructed by Youbao (Hunan, China), and the pMyc-TA-luc (#D2198-100 μg) and pRL-TK (#D2760-100 μg) plasmids were obtained from Beyotime (Shanghai, China). Transfection was performed using Jetprime (polyplus, #101000046). The lentivirus-infected cells were cultured in medium containing 2 mg/mL puromycin (Beyotime, ST551) for screening and generating stable cell lines.

### Western blot

The samples were lysed in a protein lysis buffer on ice, followed by centrifugation. The protein concentration of cell lysates was determined by Bradford reagent (Beyotime, #P0006C). The lysates were separated by SDS-PAGE and transferred to nitrocellulose membrane (Pall Corporation, #66485). The membranes were blocked and incubated with the following antibodies: anti-P21 (#2947S, CST), anti-HnRNPF (#67701-1-Ig, Proteintech), anti-GAPDH (#10442-1-AP, Proteintech), anti-α/β-Tubulin (#2148, CST), anti-EZH2 (#5246, CST), anti-H3k27me3 (#9733, CST), anti-c-Myc (#5605, CST), anti-CyclinD1 (#2978, CST). Protein bands were visualized using iBright™ CL750 Imaging System (Thermo Fisher Scientific, USA).

### Quantitative real-time PCR (qRT-PCR)

RNA was harvested using Trizol reagent (Thermo Fisher, #15596026) and then was reversed transcribed to cDNA. SYBR-green based real-time qPCR was used to assay gene expression (Trans, #AQ601), which was calculated using 2^−ΔΔCt^. Specific primers used in this experiment are shown in Table S3.

### Cell proliferation assay

The cells were seeded into a 96-well plate (2 × 10^3^/well) and cultured for 0, 24, 48, 72, and 96 h. Cells were treated with 10% CCK-8 solution (MedChemExpress, # HY-K0301) and incubated for 2 h. OD_450_ was determined using a microplate reader. For colony formation assay, 1000 cells were seeded into the 6-well plate (1 × 10^3^/well) and cultured for 8–10 days to form colonies. Cells were rinsed and fixed with 4% formaldehyde, followed by staining with 4% crystal violet. The number of cell colonies was counted with Image *J* software.

### EdU assay

Cells were labelled with 10 μM 5-Ethynyl-2′-deoxyuridine **(**EdU) and incubated at 37 °C for 2 h, then fixed with 3.7% paraformaldehyde for 15 min, then permeabilized with 0.5% Triton-X-100 for 20 min, followed by staining with Click-iT EdU Alexa Fluor 488 (ThermoFisher, #C10637). Images were acquired using an Olympus confocal microscope (FV1000, Olympus) to analyze the proportion of EdU-positive cells.

### Flow cytometry

Cells were fixed with 70% ethanol at 4 °C overnight. Cells were centrifuged and cell pellets were resuspended in PBS containing propidium iodide (Sigma, P4170). Cell cycle distribution was analyzed using a CytoFLEX flow cytometer (Beckman, USA).

### Cytoplasmic and nuclear RNA isolation

Nuclear/cytoplasmic fractionation kit (Beyotime, #P0028) was used to separate nuclear and cytoplasmic fractions. Briefly, cells were suspended in reagent A containing RNase inhibitor (Takara, #2313A) and incubated in ice bath for 15 min, followed by incubation with Reagent B in an ice bath for 1 min. The mixture was centrifuged and the supernatant represents the cytoplasmic fraction, while the sediment represents the nuclear fraction. The nuclear and cytoplasmic fractionations were lysed using Trizol Reagent (Thermo Fisher, #15596026) to extract RNA for qRT-PCR.^22^

### RNA immunoprecipitation (RIP)

RNA Immunoprecipitation was performed using MagnaRIP kit (Millipore, #17–700). Briefly, cells were lysed in RIP lysis buffer and the lysates were incubated with magnetic beads, anti-EZH2 (CST, #5246S), anti-HnRNPF (Sigma, #04–1462), or IgG antibodies. The RNA-protein immunocomplexes were incubated with RIP immunoprecipitation buffer and protein lysis buffer at 4 °C for 3 h or overnight. The immunoprecipitates were pulled down and RNA was extracted using phenol-chloroform, followed by qRT-PCR to detect the target RNA expression.

### Immunofluorescence

Cells were fixed with 4% paraformaldehyde for 15 min, then permeabilized with 0.5% Triton X-100 for 10 min. The fixed samples were blocked in 3% BSA/PBS and then incubated with primary antibodies (anti-HnRNPF, Proteintech # 67701-1-Ig, anti-EZH2, CST#5246s) at 37 °C for 1 h, followed by three washes with PBS and incubated with Alexa Fluor 488-conjugated secondary antibodies (CST, #4408S, #4412S) at 37 °C for 1 h. The cells were then stained with DAPI (Beyotime, #P0131-25 mL). Microscopy images were captured using a confocal microscope (Olympus FV1000).

### RNA *in situ* hybridization

RNA FISH of USP30-AS1 was carried out with probes designed by Stellaris™. Briefly, cells were cultured on circular cover glasses and fixed for 5 min. Further, cells were permeabilized using 0.1% Triton X-100. USP30-AS1 RNA was labeled by specific fluorescent probes at 37 °C overnight in the molecular hybridization oven. Then, cells were washed and treated with DAPI (Beyotime, #P0131-25 mL). Finally, the cells were imaged by an Olympus FV1000 microscope. The specific fluorescent probes were employed to bind to the USP30-AS1, appearing as red fluorescence under excitation light at 555 nm.

### Chromatin immunoprecipitation (ChIP)

Cells were fixed with formaldehyde and then lysed. Subsequently, the cell lysate was sonicated and centrifuged, and the supernatant was collected. Then the lysates were incubated with anti-EZH2 (CST, 5246S), anti-H3k27me3 (CST, 9733S), or anti-SPI1 (CST, 2266) antibodies, and magnetic beads (Invitrogen, #10004D) at 4 °C for 2 h. The DNA-protein complex was then washed three times using TE buffer, and DNA was purified and analyzed by real-time PCR. The primer sequences of the c-Myc promoter region are listed in Table S5.

### CRISPRa & CRISPRi

The dCAS9-VP64_GFP, pHR-SFFV-dCAS9-BFP-KRAB, pLX-sgRNA plasmids and control vector were synthesized by Youbio (Hunan, China). The sequence of each sgRNA was designed by Crispr-ERA. The sgRNA sequences of USP30-AS1 were listed in Table S6.

### RNA pull-down assay

RNA pull-down assay was performed using the MEGAscript™ T7 kit (ThermoFisher, #AM1334). Briefly, DNA with T7 promoter was transcribed *in vitro* to produce lncRNA USP30-AS1, which was further labeled with Biotin at 3′ End. The 3′ end biotin-labeled RNA was denatured and annealed. Besides, cells were lysed and the supernatant was collected. Magnetic beads (Invitrogen, #65601) were used to bind to USP30-AS1 and subsequently incubated with the supernatant for another 2 h. The Beads-USP30-AS1-proteins complex were treated with protein loading buffer (Beyotime, #P0285). Proteins that bind to USP30-AS1 were harvested and analyzed by mass spectrometry, of which results are shown in Table S7.

### Luciferase reporter assay

HEK293 cells were co-transfected with the reporter plasmid and the corresponding plasmids or siRNA using Jetprime (Polyplus, #101000046). After 48 h, cells were analyzed using the Dual-Luciferase Reporter Assay System (Promega, #E1910).

### Animal experiment

Four five-week-old female BALB/c-nude mice were purchased from Guangdong Medical Laboratory Animal Center. The mice were randomly assigned to three groups, with 5 mice in each group. The control or USP30-AS1 knockdown cells (1 × 10^6^) were subcutaneously injected into the abdominal region. The tumor size was measured using digital calipers every other day for four weeks. After four weeks, the mice were euthanized, and the tumor tissues were harvested and made into tissue sections for immunohistochemistry (IHC) and hematoxylin and eosin staining by Wuhan Servicebio Technology Co., Ltd. The experiments were approved by the Bioethics Committee of Tsinghua University Shenzhen International Graduate School (Ethics issue (2022) No. 94).

### Statistical analysis

All data were presented in terms of mean ± standard deviation values. Statistical significance was depicted as follows: ∗*p* < 0.05; ∗∗*p* < 0.01, ∗∗∗*p* < 0.001, and ∗∗∗∗*p* < 0.0001. Two-tailed Student's *t*-test and Mann–Whitney *U* test were used to compare data between two groups. The data presented here represent a minimum of three independent experiments.

## Results

### USP30-AS1 is abnormally up-regulated in breast cancer via SPI1

**Figure 1:**
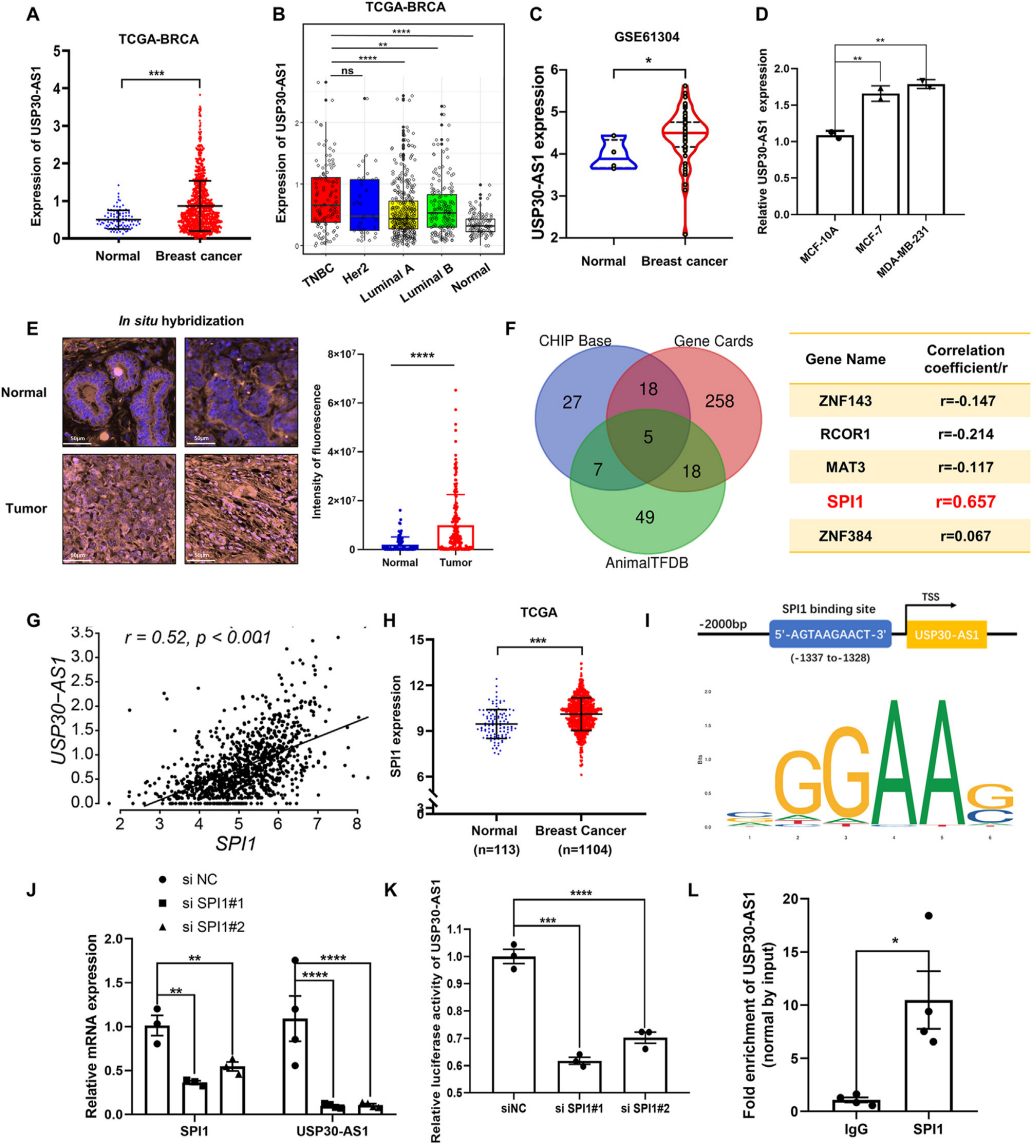
USP30-AS1 is notably up-regulated in breast cancer tissues. **(A)** The expression of USP30-AS1 in normal tissues (*n* = 113) and breast cancer tissue (*n* = 1104), and the data were obtained from the TCGA breast cancer datasets. **(B)** The expression of USP30-AS1 in various subtypes of breast cancer, including triple-negative breast Cancer (*n* = 116), HER2+ enriched breast Cancer (*n* = 37), luminal A (*n* = 375) and luminal B breast Cancer (*n* = 166). **(C)** The expression of USP30-AS1 in breast cancer tissues and normal tissues. Data were obtained from GSE61304, Mann–Whitney *U* test. **(D)** USP30-AS1 expression levels were determined by qRT-PCR in MCF10A, MCF-7, and MDA-MB-231 cell lines. **(E)** The expression of USP30-AS1 in normal breast tissues (*n* = 84) and breast cancer tissues (*n* = 176) was determined by *in situ* hybridization. **(F)** CHIP Base, Gene Cards, and Animal TFDB websites were utilized to predict the transcription factors of USP30-AS1. **(G)** Correlation analysis of SPI1 and USP30-AS1 in breast cancer tissues (*n* = 1089). **(H)** The expression levels of SPI1 in normal (*n* = 113) and breast cancer tissues (*n* = 1104), and the data were obtained from TCGA breast cancer datasets. **(I)** The binding site of SPI1 in the promoter region of USP30-AS1 was obtained from JASPAR website. **(J)** qRT-PCR showed that knockdown of SPI1 down-regulates the expression of USP30-AS1. **(K)** Dual luciferase reporter assay was conducted to assess the effect of SPI1 siRNA on transcriptional activity of the USP30-AS1. **(L)** The interaction between SPI and USP30-AS1 promoter was determined by ChIP assay. TGGA, the cancer genome atlas.

Analyzing RNA-seq data from TCGA dataset revealed significantly higher USP30-AS1 levels in breast cancer tissues compared to normal tissues (Fig. 1A). Furthermore, examination of USP30-AS1 expression across different breast cancer subtypes showed particularly elevated levels in TNBC (triple-negative breast cancer) (Fig. 1B). Gene expression analysis of the GSE61304 datasets from the GEO database also indicated abnormal up-regulation of USP30-AS1 in breast cancer patients (Fig. 1C). To validate these findings, we assessed USP30-AS1 expression in MCF-10A, MDA-MB-231, and MCF-7 cell lines, confirming higher expression in both MCF-7 and MDA-MB-231 cells compared to MCF-10A (Fig. 1D). Further analysis of the CCLE dataset proved the up-regulation of USP30-AS1 in TNBC cell lines (Fig. S1A). Additionally, *in situ* hybridization performed on tissue microarrays confirmed elevated expression of USP30-AS1 in breast cancer (Fig. 1E).

To investigate the aberrant expression of USP30-AS1 in breast cancer, we employed ChIP Base, Gene Cards, and Animal TFDB to predict the upstream transcriptional regulatory factors of USP30-AS1. Intersection analysis of the results from these three websites identified five transcription factors, including ZNF143, RCOR1, MAT3, SPI1, and ZNF384 (Fig. 1F). A significant positive correlation between USP30-AS1 and SPI1 expression (*r* = 0.52) was observed in breast cancer (Fig. 1G). A strong correlation was also observed in TNBC tissues (*r* = 0.55) (Fig. S1C, D). Moreover, an elevated expression of SPI1 in breast cancer was confirmed through RNA-seq data from the TCGA dataset (Fig. 1H). Potential SPI1-binding sites in the USP30-AS1 promoter were predicted using JASPAR (Fig. 1I; Fig. S1B). Subsequently, siRNA transfection experiments targeting SPI1 resulted in reduced expression of USP30-AS1, indicating a dependency of USP30-AS1 expression on SPI1 (Fig. 1J). Notably, dual-luciferase assays revealed a significant decrease in USP30-AS1 promoter activity following SPI1 knockdown (Fig. 1K). Furthermore, ChIP assay demonstrated the binding between the SPI1 and USP30-AS1 promoter (Fig. 1L). In summary, these results indicate that SPI1 may contribute to the up-regulation of USP30-AS1 in breast cancer.

### USP30-AS1 plays an oncogenic role in breast cancer

**Figure 2:**
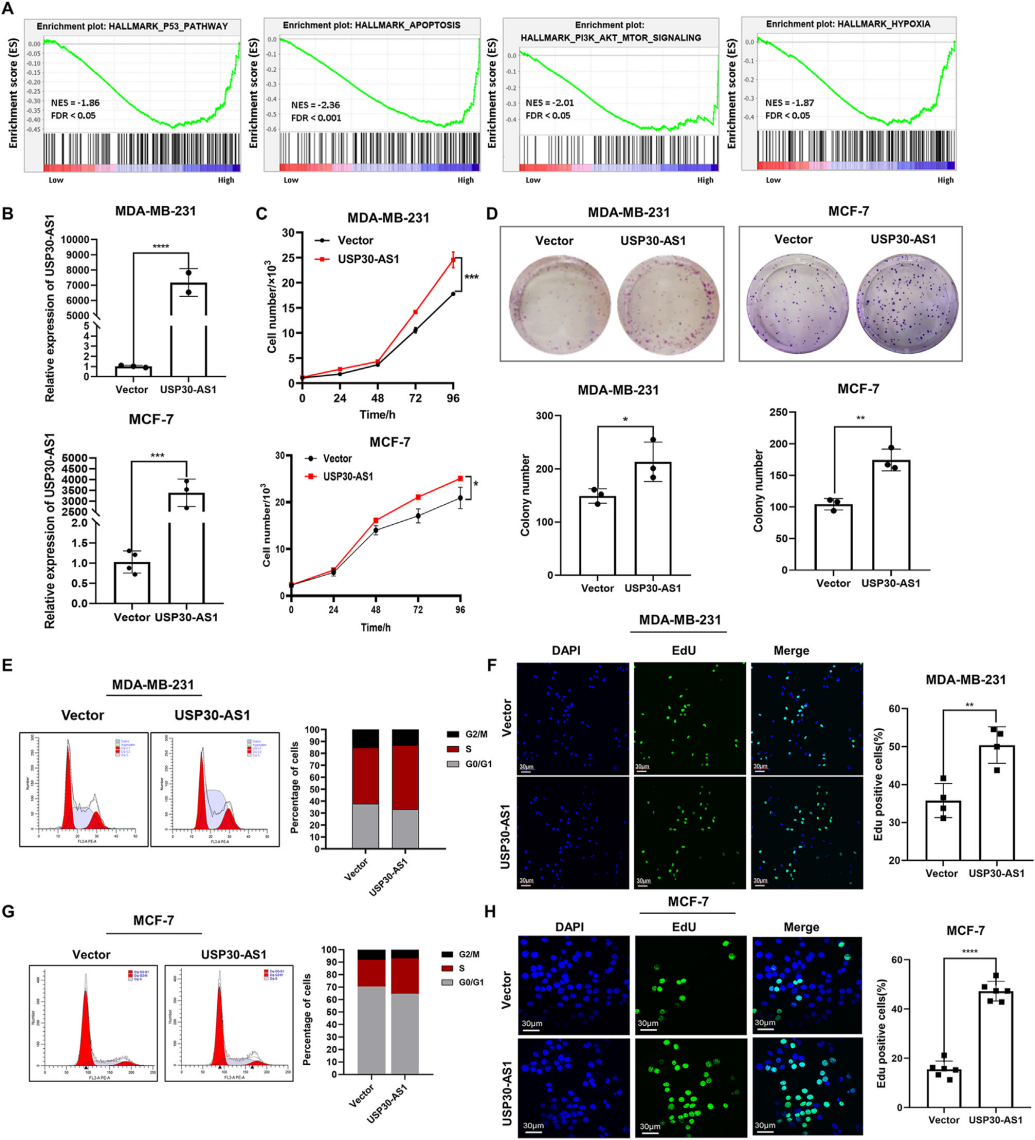
Overexpression of USP30-AS1 promotes breast cancer cell proliferation. **(A)** Gene Set Enrichment Analysis (GSEA) revealed USP30-AS1 is involved in proliferation-related signaling pathways. **(B)** The overexpression efficiency of USP30-AS1 was validated by qRT-PCR. **(C)** The effects of USP30-AS1 overexpression on the proliferation of MDA-MB-231 and MCF-7 cells were analyzed by CCK8 assay. **(D)** The effect of USP30-AS1 overexpression on the proliferation capacity of MDA-MB-231 and MCF-7 cells was evaluated by colony formation assays. **(E, G)** Flow cytometry analysis and statistical analysis were conducted to determine the impact of USP30-AS1 overexpression on cell cycle progression in MDA-MB-231 and MCF-7 cells. **(F, H)** EdU assays were performed to determine the effects of USP30-AS1 on DNA replication in MDA-MB-231 and MCF-7 cells.

We conducted Gene Set Enrichment Analysis (GSEA) to explore the potential function of USP30-AS1 in breast cancer. The results revealed that high level of USP30-AS1 is correlated with the PI3K-AKT-MTOR, APOPTOSIS, HYPOXIA, and P53 signaling pathways, suggesting that USP30-AS1 might be involved in regulating cell proliferation (Fig. 2A). To validate the bioinformatics analysis results, we overexpressed USP30-AS1 in MCF-7 and MDA-MB-231 cells (Fig. 2B). Cell proliferation assays showed that ectopic expression of USP30-AS1 promoted cell proliferation and colony formation (Fig. 2C, D). Additionally, flow cytometry experiments showed that USP30-AS1 overexpression markedly increased the S phase cell population, indicating its involvement in cell cycle regulation (Fig. 2E–G). To further analyze the impact of USP30-AS1 on DNA replication, we used an EdU imaging kit to measure the incorporation of EdU into DNA during DNA synthesis. Consistently, exogenous USP30-AS1 markedly increased the S phase cell population (Fig. 2F–H).

### Knockdown of USP30-AS1 suppresses breast cancer cell progression

**Figure 3:**
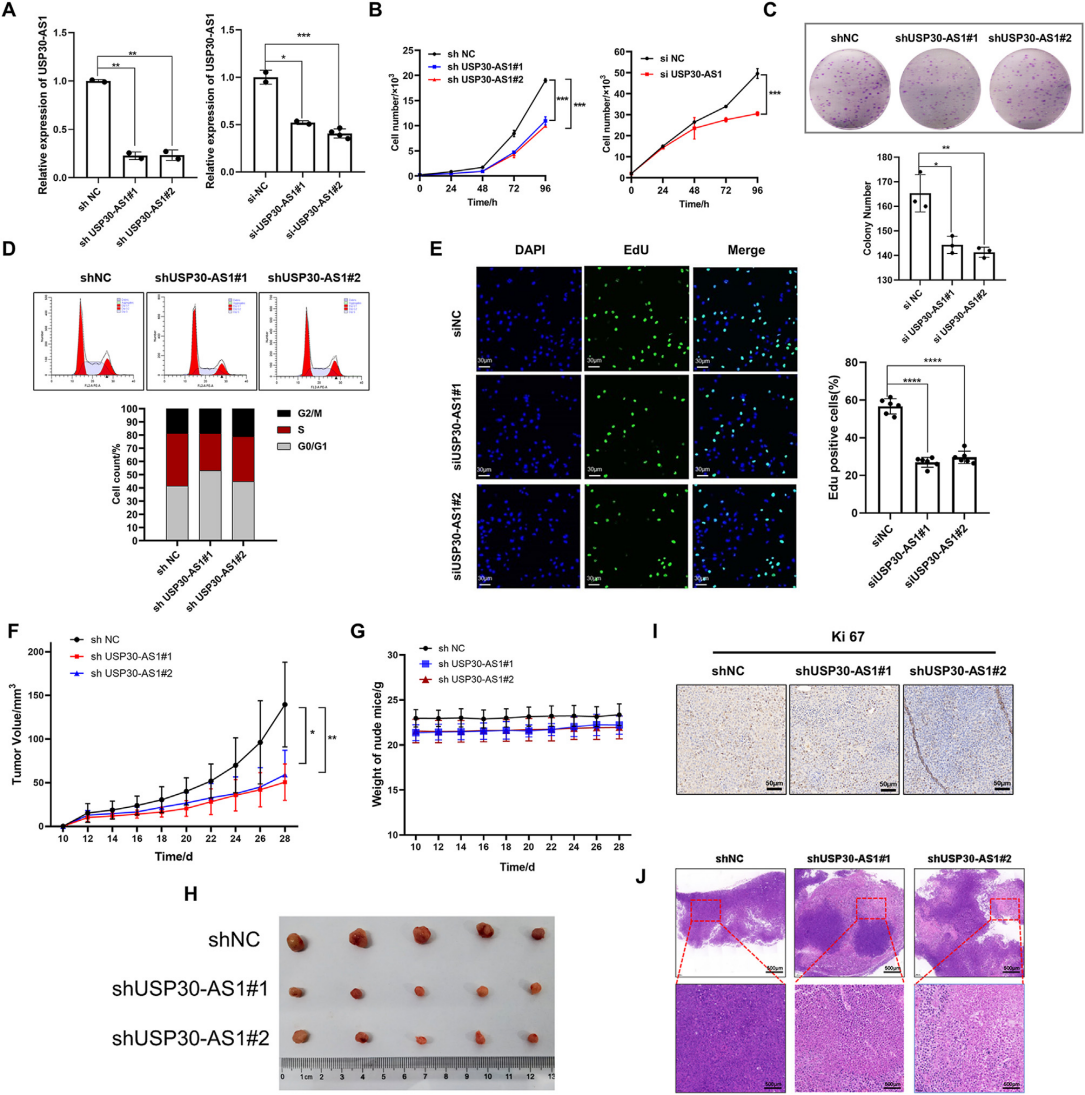
USP30-AS1 knockdown inhibits breast cancer cell proliferation both *in vitro and in vivo*. **(A)** The knockdown efficacy of USP30-AS1 siRNA and shRNA was validated by qRT-PCR analysis. **(B)** The effect of USP30-AS1 knockdown on MDA-MB-231 cell proliferation was evaluated by CCK-8 assays. **(C)** The impact of USP30-AS1 knockdown on the proliferative capacity of MDA-MB-231 cells was assessed by colony formation assays. **(D)** Flow cytometry analysis was performed to determine the effect of USP30-AS1 knockdown on the cell cycle progression of MDA-MB-231 cells. **(E)** EdU assays were conducted in MDA-MB-231 cells with USP30-AS1 knockdown. **(F)** A xenograft tumor experiment was conducted using three MDA-MB-231 cell lines: shNC, shUSP30-AS1#1, and shUSP30-AS1#2. **(G)** Tumor growth and tumor weight were assessed every two days. **(H)** The actual photo of tumors for each group was collected at the end of the experiment. **(I)** Hematoxylin and eosin staining of tumor tissues from nude mice. **(J)** Immunohistochemistry assays were performed to determine the impact of USP30-AS1 knockdown on Ki67 expression within xenograft tumor tissues.

Next, we employed siRNA or shRNA-mediated gene silencing strategies to deplete USP30-AS1 in MCF-7 and MDA-MB-231 cells (Fig. 3A; Fig. S2A). Cell proliferation assays demonstrated that USP30-AS1 knockdown significantly attenuated cell proliferation (Fig. 3B, C; Fig. S2B, C). Additionally, flow cytometry experiments revealed that depletion of USP30-AS1 reduced the number of S phase cells (Fig. 3D; Fig. S2D). Furthermore, using an EdU imaging kit to assess DNA synthesis, a marked reduction of EdU-positive cells was observed upon siRNA-mediated USP30-AS1 knockdown (Fig. 3E; Fig. S2E).

Previous studies suggested that cell cycle arrest can lead to apoptotic cell death.^23^ Therefore, we conducted Annexin V/PI assay to analyze the impact of USP30-AS1 on apoptosis. Depletion of USP30-AS1 promoted apoptosis, while ectopic expression of USP30-AS1 suppressed apoptotic cell death (Fig. S3). Additionally, we observed an acceleration of cellular senescence following USP30-AS1 knockdown using β-galactosidase assays (Fig. S4).

To validate the role of USP30-AS1 in breast cancer progression *in vivo*, a xenograft tumor experiment was performed in nude mice. Knockdown of USP30-AS1 markedly reduced tumor growth and progression (Fig. 3F–H). The control group exhibited tumor parenchyma with a small amount of stroma and densely packed cell arrangements. In contrast, the shUSP30-AS1 group displayed more pronounced nuclear dissolution and extensive areas of cell necrosis (Fig. 3I). Additionally, IHC analysis of tumor tissues demonstrated a reduction in Ki67 expression following USP30-AS1 knockdown (Fig. 3J). These results demonstrate that USP30-AS1 promotes breast cancer proliferation both *in vitro* and *in vivo*.

### USP30-AS1 promotes cell proliferation through the down-regulation of p21

**Figure 4:**
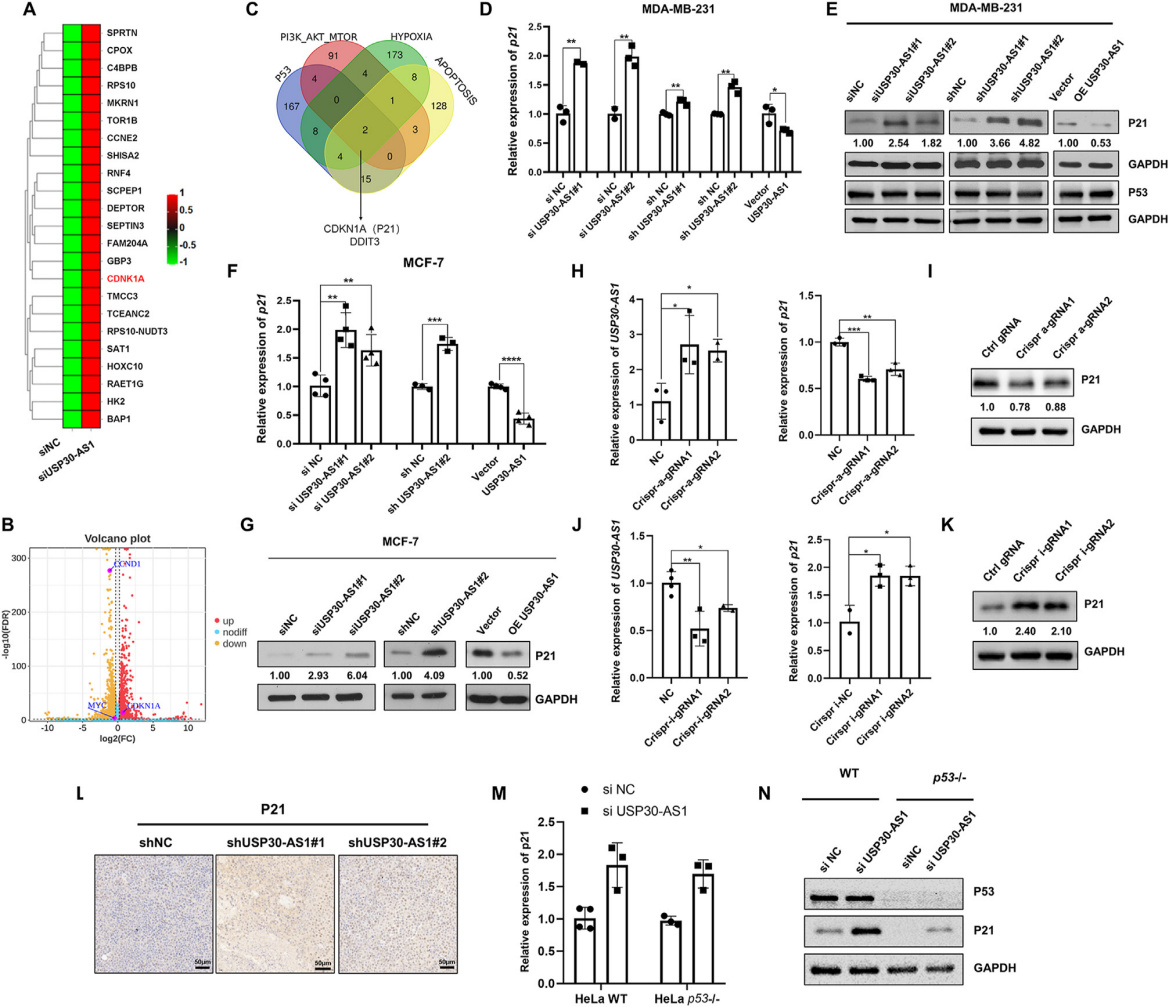
USP30-AS1 promotes breast cancer cell proliferation by suppressing the expression of p21. **(A)** The differentially expressed genes in control and USP30-AS1 knockdown cells were identified by RNA-seq analysis. **(B)** Volcano plot of RNA-seq after USP30-AS1 knockdown highlighting CDKN1A/p21. **(C)** The overlapping genes of PI3K-AKT, hypoxia, apoptosis, and P53 signaling pathways that are predicted by GSEA. **(D, F)** qRT-PCR and **(E, G)** Western blot analysis was conducted in MDA-MB-231 and MCF-7 cells with USP30-AS1 knockdown or overexpression. **(H)** qRT-PCR and **(I)** Western blot analysis showed that CRISPR-Cas activator (CRISPRa) induces up-regulation of USP30-AS1 and down-regulation of p21. **(J)** qRT-PCR and **(K)** Western blot analysis demonstrated that CRISPR interference (CRISPRi) suppresses USP30-AS1 expression and up-regulates p21 expression. **(L)** The tumor tissues from nude mice were subjected to immunohistochemistry to determine the expression of P21 in each group. **(M)** USP30-AS1 expression was knocked down in wild type and *p53*^*−/−*^ HeLa cells; the mRNA and **(N)** protein expression of p21 were analyzed by qPCR and Western blot.

To study the molecular mechanism by which USP30-AS1 regulates breast cancer cell proliferation, we employed transcriptome sequencing in control and USP30-AS1 knockdown cells. RNA-seq and GSEA analyses revealed that silencing USP30-AS1 altered the expression of multiple proliferation-associated genes, including CDKN1A/p21, a well-known cell cycle inhibitor (Fig. 4A–C). Therefore, we investigated whether USP30-AS1 regulates cell proliferation by modulating the expression of p21. Both qRT-PCR and immunoblotting results indicated that knockdown of USP30-AS1 markedly increased the expression of p21, whereas overexpression of USP30-AS1 suppressed the expression of p21 in MCF-7 and cells. The expression of p53, the well-known upstream transcription factor of p21, remained unchanged upon USP30-AS1 silencing or overexpression (Fig. 4D–G). Additionally, we employed CRISPR activation (CRISPRa) and CRISPR interference (CRISPRi) techniques to manipulate USP30-AS1 transcription. HEK293 cells transfected with dCas9-VP64 and sgRNAs to transactivate endogenous USP30-AS1 exhibited a significant decrease in p21 levels (Fig. 4H, I). Conversely, CRISPRi-mediated genetic silencing of USP30-AS1 increased p21 expression (Fig. 4J, K).

Furthermore, IHC experiments were conducted on tumor tissues obtained from previous animal experiments. Mice with USP30-AS1 knockdown exhibited increased p21 intensity, further supporting the notion that USP30-AS1 promotes breast cancer progression by down-regulating p21 (Fig. 4L).

It has been reported that the transcription of p21 is regulated through both p53-dependent and p53-independent mechanisms.^24^ To determine whether p53 is involved in the regulation of p21 by USP30-AS1, we knocked down USP30-AS1 in both wild type and *p53*^*−/−*^ cells. qPCR and immunoblotting results indicated that p53 did not influence the siUSP30-AS1-mediated up-regulation of p21, suggesting that USP30-AS1 regulates p21 expression through a p53-independent mechanism (Fig. 4M, N).

To validate the independence of USP30-AS1 from its neighboring gene *USP30,* we assessed the protein levels of *USP30* in MDA-MB-231 and HEK293 following USP30-AS1 knockdown or overexpression. The results revealed no significant changes in *USP30* expression, confirming that the functional role of USP30-AS1 in breast cancer is independent of *USP30*. Therefore, the observed phenotypes can be attributed to USP30-AS1 itself rather than indirect regulation by *USP30* (Fig. S5C, D).

### USP30-AS1 regulates p21 mRNA stability through HnRNPF

**Figure 5:**
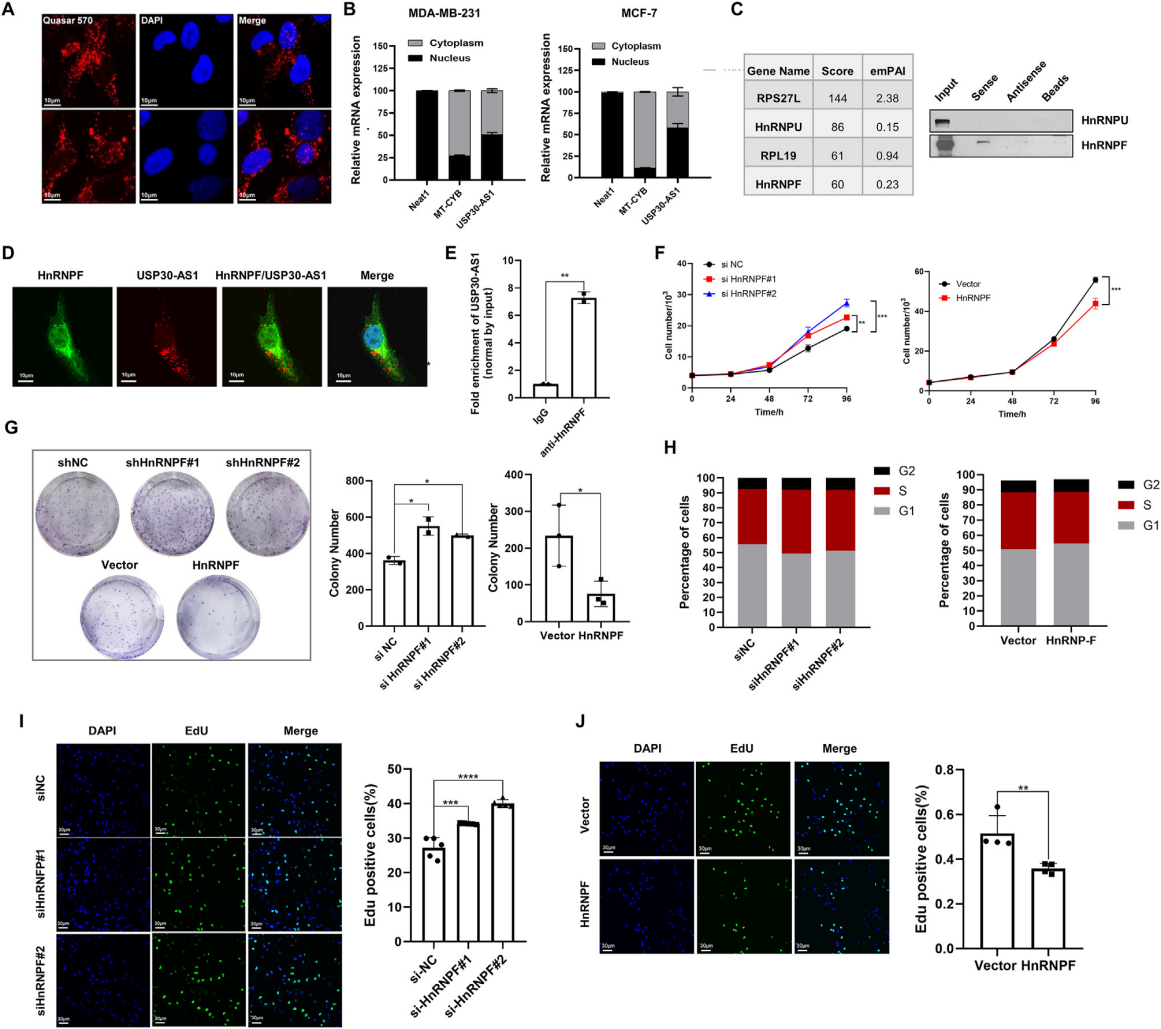
USP30-AS1 binds to HnRNPF and HnRNPF regulates breast cancer cell proliferation. **(A)** FISH assay was conducted to determine the localization of USP30-AS1 in MDA-MB-231 cells. The representative images were acquired using confocal microscopy. **(B)** Nuclear-cytoplasmic fractionation was performed in MDA-MB-231 and MCF-7 cells. The expression of USP30-AS1 in the nucleus and cytoplasm was evaluated by qRT-PCR. Neat1 and MT-CYB were used as markers for the nuclear and cytoplasmic localized genes, respectively. **(C)** The predicted binding scores of USP30-AS1 with HnRNPF, RPS2L, RPL19, and HnRNP-U proteins based on mass spectrometry results. The results of RNA pull-down assay in MDA-MB-231 cells confirmed the interaction between USP30-AS1 and HnRNPF. **(D)** Confocal images showed the colocalization of USP30-AS1(red) and HnRNPF (green) in MDA-MB-231 cells. **(E)** RIP assay showed the interaction between HnRNPF and USP30-AS1 in MDA-MB-231 cells. **(F)** The effect of HnRNPF on breast cancer cell proliferation was conducted in MDA-MB-231 cells transfected with HnRNPF siRNA or HnRNPF plasmid. **(G)** The impact of HnRNPF on the proliferation capacity of MDA-MB-231 cells was evaluated by colony formation assays. **(H)** Flow cytometry analysis was conducted to determine the impact of HnRNPF on cell cycle progression in MDA-MB-231 cells. **(I)** EdU assays were performed in MDA-MB-231 cells with HnRNPF knockdown or **(J)** overexpression, and the percentage of EdU positive cells was quantified based on EdU staining.

LncRNAs are spatially compartmentalized within cells, a feature that is closely linked to their biological functions and cellular activities. Our RNA FISH assay revealed that USP30-AS1 exhibits dual localization in both the cytoplasm and nucleus (Fig. 5A). Cell fractionation assay further confirmed dual localization of USP30-AS1 (Fig. 5B). Knockdown of USP30-AS1 reduced the expression of endogenous USP30-AS1 in both cytoplasm and nucleus, whereas overexpression had the opposite effect (Fig. S6A, B).

LncRNAs possess the ability to interact with DNA, RNA, and proteins, thereby modulating gene expression at multiple levels.^25^ To identify proteins that specifically binds to USP30-AS1, we performed RNA pull-down assays followed by mass spectrometry, identifying 69 proteins localized in the cytoplasm, nucleus, and other cellular compartments (Fig. S6C and Table S7). Notably, proteins like RPL19, RPS27L, HnRNPF, and HnRNPU involved in pre-mRNA splicing and protein translation processes were identified. The physical interaction between USP30-AS1 and HnRNPF was further confirmed by RNA pull-down and Western blot analysis (Fig. 5C). FISH combined with immunofluorescence assays further confirmed the colocalization of USP30-AS1 and HnRNPF in the cytoplasm (Fig. 5D). Additionally, RNA immunoprecipitation assay revealed specific interaction between HnRNPF and USP30-AS1 (Fig. 5E). The subcellular localization of HnRNPF was determined by nuclear/cytoplasmic extraction followed by Western blot. HnRNPF distributed in both cytoplasm and nucleus, akin to USP30-AS1 (Fig. S6D).

To study the function of HnRNPF in breast cancer, we analyzed the top 1000 genes highly correlated with HnRNPF using the Metascape website for functional enrichment analysis. The results suggested that HnRNPF may regulate DNA damage response, RNA metabolism, and the cell cycle (Fig. S6E). CCK-8 and colony formation assays indicated that depletion of HnRNPF enhanced cell proliferation, whereas overexpression of exogenous HnRNPF had a repressive effect on cell proliferation and colony formation (Fig. 5F, G). Flow cytometry and EdU assays further revealed that depletion of HnRNPF promoted DNA replication, whereas overexpression of HnRNPF showed the opposite effect (Fig. 5H–J).

**Figure 6:**
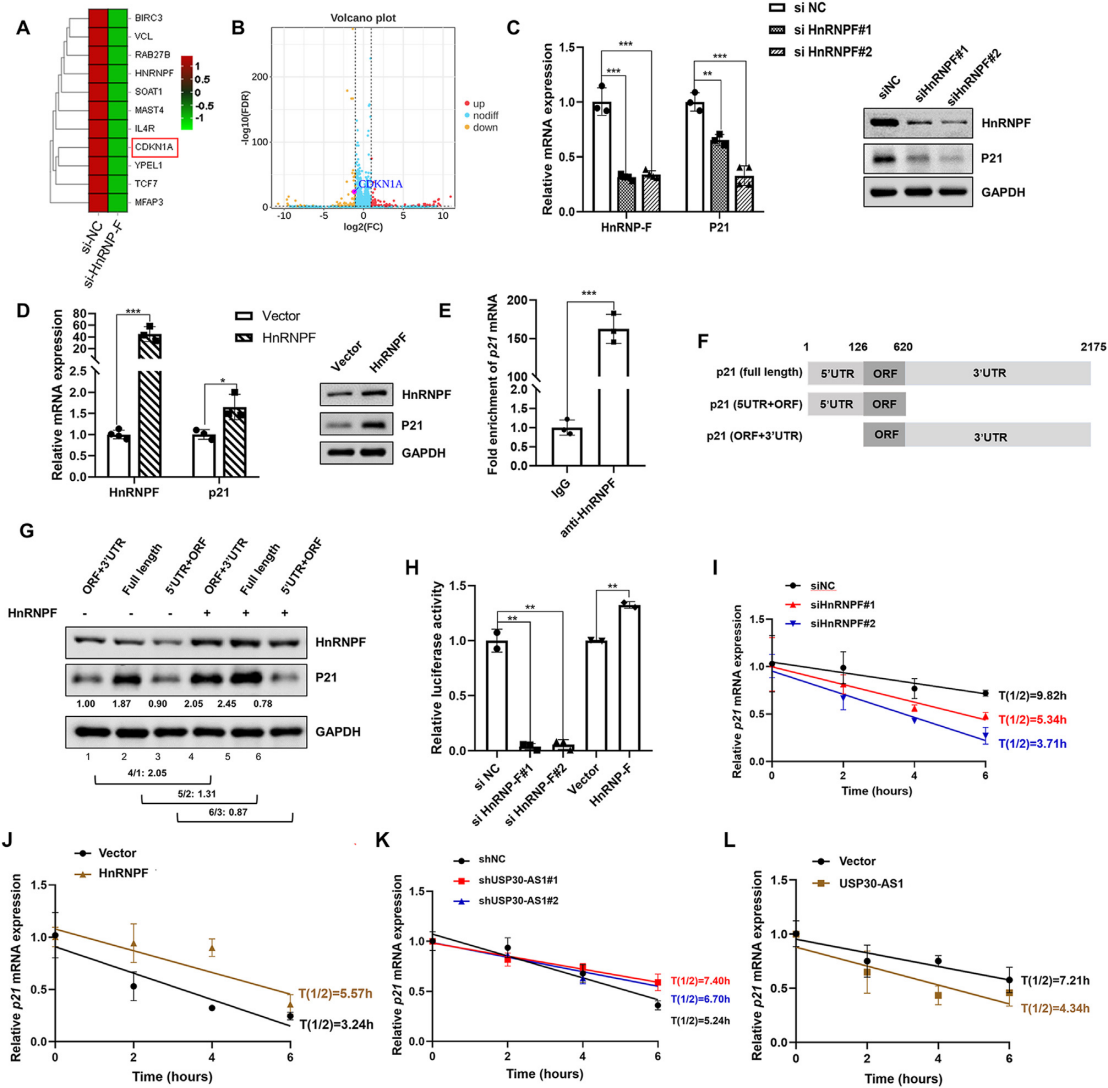
USP30-AS1 and HnRNPF regulate p21 mRNA stability. **(A)** The differentially expressed genes in control and HnRNPF knockdown cells were identified by RNA-seq analysis. **(B)** Volcano plot of RNA-seq after HnRNPF knockdown highlighting CDKN1A/p21**. (C)** qPCR and Western blot analysis showed that HnRNPF knockdown or **(D)** overexpression modulates the expression of p21. **(E)** RIP assay was performed to confirm the interaction between HnRNPF and P21 mRNA in MDA-MB-231 cells. **(F)** The expression vectors contain the p21 coding region (Open Reading Frame) in combination with either the 3′UTR, the 5′UTR, or both. **(G)** The protein levels of p21 were determined by Western blot analysis. **(H)** Luciferease reporter assay was conducted in HEK293 cells transfected with reporter containing the full-length 3′UTR. **(I)** The effects of USP30-AS1 knockdown or **(J)** overexpression on the half-life of p21 mRNA were assessed by qRT-qPCR after treatment with actinomycin D (10 μg/mL) for different time points **(K)** The effects of HnRNPF knockdown or **(L)** overexpression on the half-life of p21 mRNA were analyzed by qRT-qPCR after treatment with actinomycin D.

CDKN1A/p21 was found to be down-regulated upon HnRNPF silencing through RNA-seq analyses (Fig. 6A, B). qPCR and immunoblotting further validated this finding (Fig. 6C). Conversely, exogenous HnRNPF increased p21 expression in breast cancer cells (Fig. 6D; Fig. S6F). RIP assay revealed the interaction between HnRNPF and p21 mRNA (Fig. 6E). It has been reported that HnRNP family act as an RNA scaffold, recruiting mRNA to affect its splicing and processing as well as regulating gene transcription and translation.^26^ Next, we constructed expression vectors containing the p21 coding region (Open Reading Frame) along with either the 3′UTR, the 5′UTR, or both.^27^ Western blot revealed that HnRNPF significantly increased p21 protein level in cells transfected with either full-length p21 or p21 containing the 3′UTR, indicating that HnRNPF regulates p21 expression through its 3′UTR region (Fig. 6F, G). The dual-luciferase reporter assay revealed that knockdown of HnRNPF significantly decreased the luciferase activity of p21 3′UTR, whereas exogenous HnRNPF had the opposite effect (Fig. 6H).

RNA-binding proteins typically regulate mRNA stability by binding to the 3′UTR.^28^^,^^29^ To examine whether HnRNPF plays a role in regulating p21 mRNA stability, we treated MDA-MB-231 cells with actinomycin D to block *de novo* RNA synthesis. Knockdown of HnRNPF accelerated the decay of p21 mRNA, whereas exogenous HnRNPF prolonged its half-life, indicating that HnRNPF binds to and stabilizes p21 mRNA (Fig. 6I, J). Subsequently, we assessed the impact of USP30-AS1 on p21 mRNA stability using similar experiment. Remarkably, depletion of USP30-AS1 delayed p21 mRNA degradation, while its overexpression expedited p21 mRNA decay (Fig. 6K, L). These findings suggested opposite roles of USP30-AS1 and HnRNPF in p21 mRNA stability.

**Figure 7:**
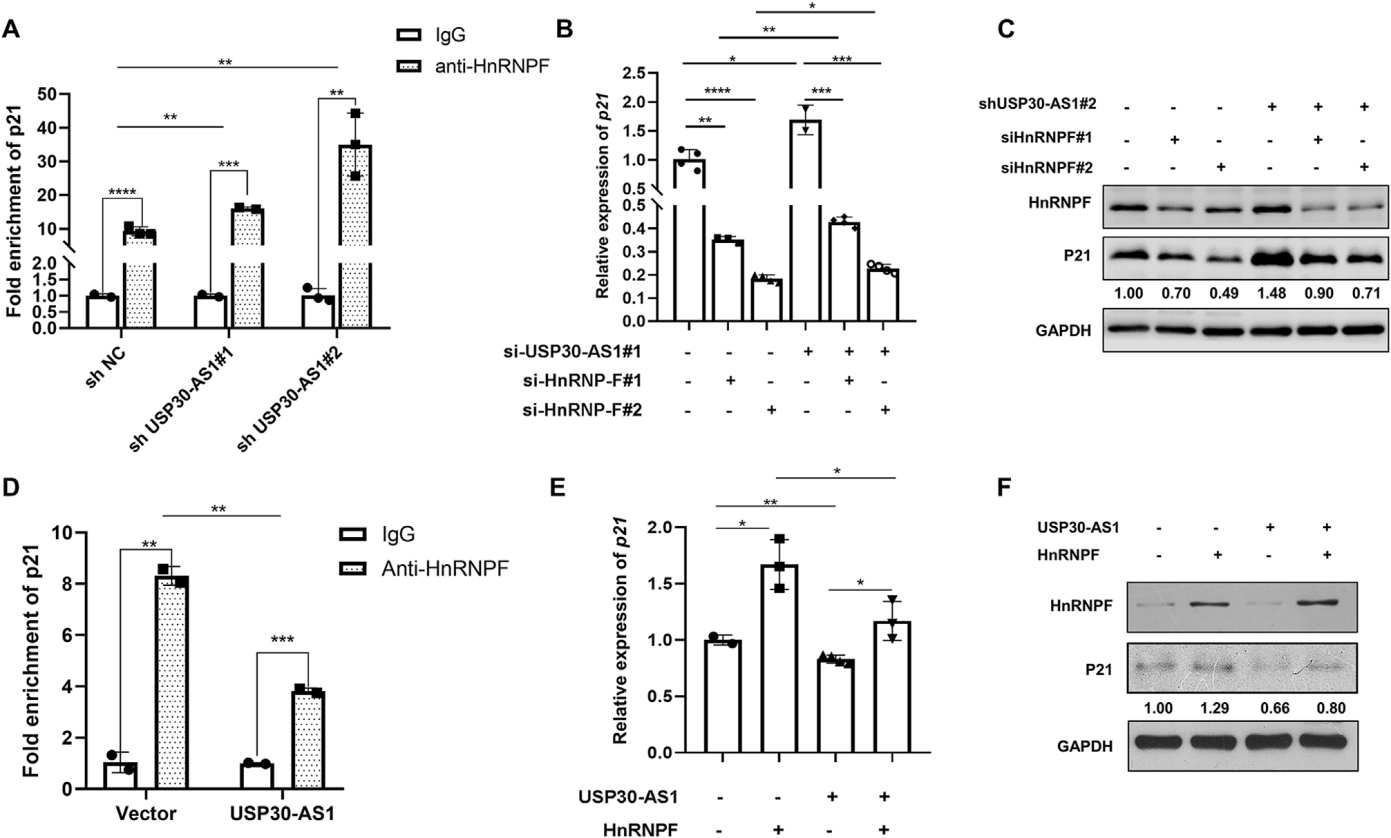
USP30-AS1 reverses the regulatory effect of HnRNPF on p21. **(A)** RIP assay showed that knockdown of USP30-AS1 enhanced the interaction between HnRNPF and p21 mRNA. **(B)** qRT-PCR and **(C)** Western blot analysis was conducted to analyze the impact of USP30-AS1 shRNA on HnRNPF knockdown-mediated p21 down-regulation in MDA-MB-231 cells. **(D)** RIP assay demonstrated that overexpression of HnRNPF blocked the interaction between HnRNPF and p21 mRNA. **(E)** qRT-PCR and **(F)** Western blot analysis was conducted to analyze the impact of USP30-AS1 overexpression on HnRNPF-mediated up-regulation of p21 in MDA-MB-231 cells.

We hypothesized that USP30-AS1 may act as a decoy to interfere with HnRNPF's impact on p21 mRNA stability. RIP experiments proved that knockdown of USP30-AS1 dramatically increased HnRNPF enrichment at p21 mRNA (Fig. 7A). Subsequent qPCR and Western blot confirmed that depletion of USP30-AS1 restored p21 expression in HnRNPF knockdown cells (Fig. 7B, C). Consistently, overexpression of USP30-AS1 disrupted HnRNPF binding to p21 mRNA (Fig. 7D). Moreover, USP30-AS1 attenuated the regulatory effect of HnRNPF on p21 expression (Fig. 7E, F). Therefore, these results demonstrate that USP30-AS1 regulates p21 mRNA stability through its interaction with HnRNPF.

### USP30-AS1 regulates p21 transcription through c-myc

**Figure 8:**
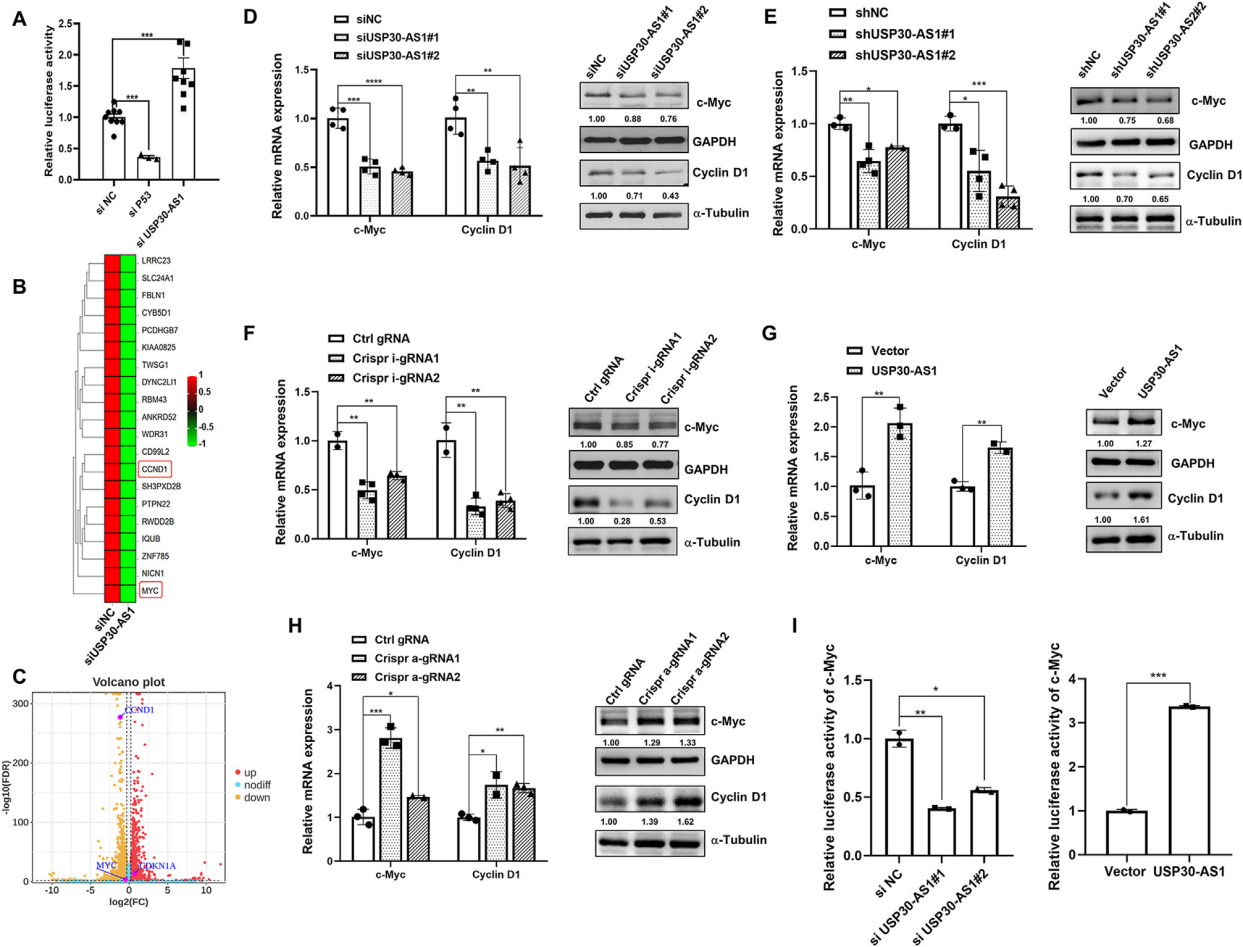
USP30-AS1 regulates the expression of c-Myc and cyclin D1. **(A)** Dual luciferase reporter assays were performed to assess the effect of USP30-AS1 and p53 on the transcriptional activity of p21. **(B)** RNA-seq analysis was employed to identify the genes exhibiting differential expression following knockdown of USP30-AS1. **(C)** Volcano plot of RNA-seq after USP30-AS1 knockdown highlighting MYC and CCND1. **(D)** Knockdown of USP30-AS1 by siRNA or **(E)** shRNA modulated the expression of c-Myc and cyclin D1. **(F)** The effect of CRISPRi-mediated USP30-AS1 inactivation on the expression of c-Myc and cyclin D1 was analyzed by qPCR and Western blot. **(G)** Overexpression of USP30-AS1 up-regulated the expression of c-Myc and cyclin D1 in MDA-MB-231 cells. **(H)** The effect of CRISPRa-mediated USP30-AS1 activation on the expression of c-Myc and cyclin D1 was analyzed by qPCR and Western blot. **(I)** The impact of USP30-AS1 on the transcriptional activity of c-Myc was validated by dual luciferase reporter assays.

We employed a luciferase-based reporter system to monitor the transcriptional activity of p21 promoter. The results suggested that USP30-AS1 silencing increased p21 transcription, implying that USP30-AS1 also regulated p21 expression at the transcriptional level (Fig. 8A). RNA-seq analysis revealed that depletion of USP30-AS1 reduced the expression of several genes, including MYC and CCND1 (Fig. 8B, C). Given previous reports of c-Myc as a transcriptional repressor of p21,^30^ we hypothesized that USP30-AS1 might also regulate p21 transcription through c-Myc. To validate this, we performed qPCR and immunoblotting to assess the relationship between USP30-AS1 and c-Myc. Knockdown of USP30-AS1 via siRNA, shRNA, or CRISPRi significantly suppressed the expression of c-Myc and Cyclin D1 (Fig. 8D–F; Fig. S7A). Conversely, overexpression of USP30-AS1 increased c-Myc and Cyclin D1 expression (Fig. 8G, H; Fig. S7B). We constructed a dual-luciferase reporter containing the c-Myc promoter to examine the role of USP30-AS1 on c-Myc transcription. Knockdown of USP30-AS1 reduced the transcriptional activity of the c-Myc promoter, while overexpression of USP30-AS1 increased it (Fig. 8I). These results demonstrated that USP30-AS1 could transcriptionally repress p21 expression through c-Myc.

### USP30-AS1 epigenetically regulates c-myc expression through EZH2

**Figure 9:**
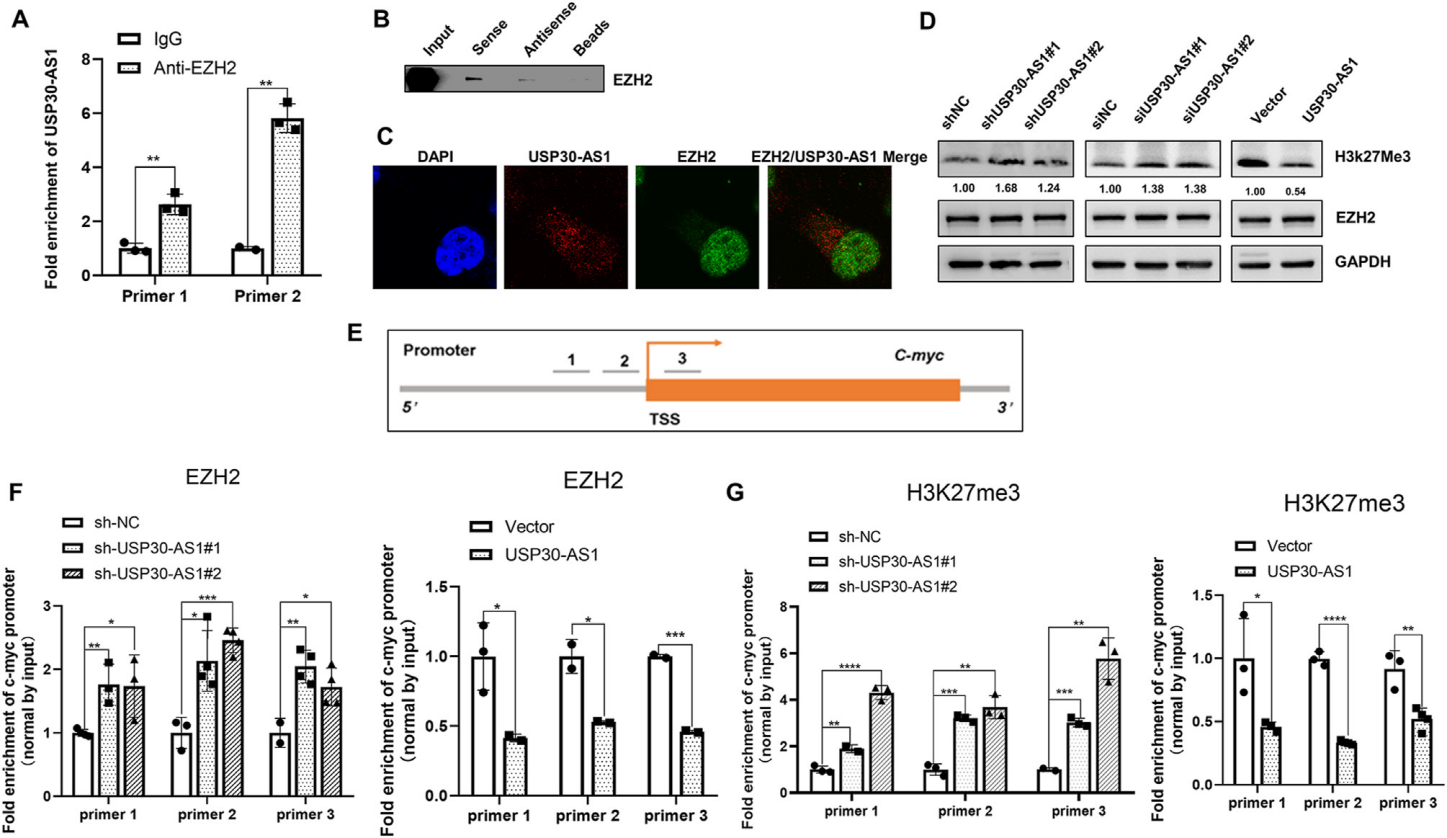
USP30-AS1 interacts with EZH2 to epigenetically regulate c-Myc expression. **(A)** RIP assay and qRT-PCR were conducted to investigate the interaction between EZH2 and USP30-AS1 in MDA-MB-231 cells. **(B)** RNA pull-down and Western blot confirmed the interaction between HnRNPF and USP30-AS1 in MDA-MB-231 cells. **(C)** Confocal images showed the colocalization of USP30-AS1 (red) and EZH2 (green) in MDA-MB-231 cells. **(D)** The effect of USP30-AS1 on the expressions of EZH2 and H3k27me3 was determined by Western blot. **(E)** Three primer pairs were designed near the c-Myc TSS for ChIP assay. **(F)** The effect of USP30-AS1 on the level of H3K27me3 at the c-Myc promoter was detected by ChIP-qPCR assay in MDA-MB-231 cells. **(G)** The impact of USP30-AS1 on the recruitment of EZH2 to the c-Myc promoter was assessed by ChIP-qPCR assay in MDA-MB-231 cells.

The c-Myc oncogene is overexpressed in human breast cancer and is linked to poor prognosis.^31^ To elucidate the molecular mechanisms by which USP30-AS1 affects c-Myc transcription, we utilized CatRAPID and RPISeq platforms to predict interactions between lncRNA and proteins. These analyses identified EZH2 as a potential binding protein for USP30-AS1(Fig. S9A, B). Subsequent RNA pull-down and RIP assays confirmed that USP30-AS1 physically interacts with EZH2 (Fig. 9A, B). Moreover, EZH2 was found to colocalize with USP30-AS1 in the nucleus of breast cancer cells (Fig. 9C).

EZH2 is the enzymatic catalytic component of the polycomb repressive complex 2 (PRC2), responsible for catalyzing the trimethylation of histone H3 at lysine K27 (H3K27me3), a modification that promotes chromatin condensation and gene silencing.^32^ Immunoblotting revealed that knockdown of USP30-AS1 increases the level of H3K27me3, whereas overexpression of USP30-AS1 substantially reduced H3K27me3 level (Fig. 9D). To determine the effect of USP30-AS1 on H3K27me3 and EZH2 enrichment at the c-Myc promoter region, we designed primer pairs targeting regions upstream and downstream of c-Myc transcription start site (TSS) and performed chromatin immunoprecipitation (ChIP) experiment (Fig. 9E). ChIP-qPCR results demonstrated that USP30-AS1 silencing significantly elevated the enrichment of H3K27me3 and EZH2 near the c-Myc TSS. Conversely, overexpression of USP30-AS1 noticeably reduced the enrichment of H3K27me3 and EZH2 at the c-Myc promoter (Fig. 9F, G). These results suggest that USP30-AS1 impedes the recruitment of EZH2 to the c-Myc promoter, thereby reducing H3K27 trimethylation occupancy and epigenetically up-regulating c-Myc expression.

## Discussion

Extensive evidence indicates that the progression of breast cancer is strongly associated with dysregulation of lncRNA expression. Herein, we identify USP30-AS1 as novel oncogenic lncRNA markedly up-regulated in breast cancer tissues and cell lines. While USP30-AS1 exhibits context-dependent tumor-promoting or suppressive roles across cancers, its functional significance and molecular mechanisms in breast cancer remained unclear. Our study reveals that USP30-AS1 drives breast cancer progression through dual cytoplasmic and nuclear mechanisms. In the cytoplasm, USP30-AS1 directly binds to HnRNPF, disrupting p21 mRNA stability. While in the nucleus, USP30-AS1 interacts with EZH2, reducing H3K27 trimethylation of the c-Myc promoter, this epigenetic reprogramming elevates c-Myc transcription, which further represses p21 expression. Notably, although its host gene USP30 has been reported to drive breast cancer progression by stabilizing Snail,^33^ the function of USP30-AS1 is independent of USP30, suggesting that antisense transcripts from the same gene locus may participate in cancer regulation through distinct mechanisms.

We performed transcriptome sequencing combined with integrated bioinformatic analysis, through which we identified p21, c-Myc, and cyclin D1 as downstream targets of USP30-AS1. Knockdown of USP30-AS1 using siRNA, shRNA or CRISPRi strategies resulted in up-regulation of p21 and down-regulation of c-Myc and cyclin D1. USP30-AS1 specifically interacts with HnRNPF, a member of HnRNP RNA-binding protein family known to regulate various stages of RNA metabolism.^34^^,^^35^ HnRNPF regulates gene expression through various mechanisms, including mRNA stability and alternative splicing. For instance, HnRNPF can bind to the 3′UTR of Snail mRNA, enhancing its stability and thereby influencing the epithelial–mesenchymal transition in bladder cancer.^26^^,^^36^ Additionally, HnRNPF can inhibit the migration and invasion of breast cancer cells, and patients with higher HnRNPF expression exhibited a better prognosis.^37^ In this study, we demonstrate that USP30-AS1 interacts with HnRNPF, preventing its binding to the p21 3′UTR. This interaction resulted in p21 mRNA destabilization and down-regulation of p21 protein expression. Depletion of USP30-AS1 enhanced the interaction between HnRNPF and p21 mRNA, increasing p21 mRNA stability and ultimately inhibiting breast cancer cell proliferation.

The CDK inhibitor p21 is transcriptionally regulated by multiple signaling pathways and transcription factors. Various stimuli can activate p21 through either p53-dependent or p53-independent mechanisms, resulting in growth arrest and cellular senescence. The transcriptional repression of p21 by c-Myc plays a critical role in cancer development.^38^ Our results suggest that USP30-AS1 not only reduces p21 mRNA stability via HnRNPF but also transcriptionally represses p21 through a p53-independent mechanism. Specifically, USP30-AS1 binds to EZH2, which regulates the transcription of downstream target genes by modulating H3K27me3.^39^^,^^40^ USP30-AS1 prevents EZH2 from binding to the c-Myc promoter, leading to epigenetic up-regulation of c-Myc expression by reduced H3k27me3 levels. The up-regulation of c-Myc suppresses p21 transcription and further accelerates breast cancer cell proliferation.

**Figure 10:**
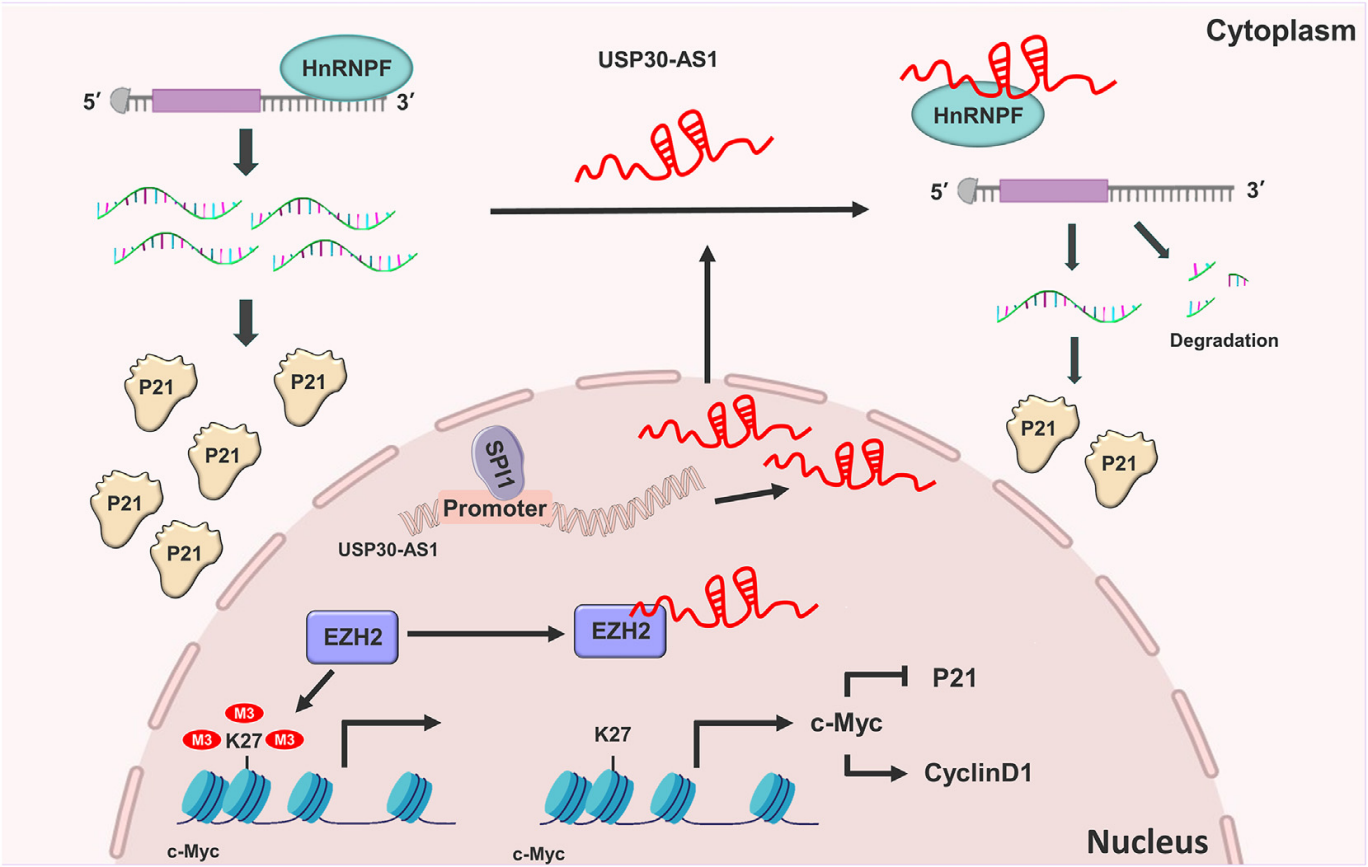
The schematic diagram illustrating that nuclear and cytoplasmic coordinately regulate breast cancer progression. In the cytoplasm, USP30-AS1 interacts with HnRNPF to prevent the binding between HnRNPF and p21 mRNA, resulting in the destabilization of p21 mRNA. In the nucleus, USP30-AS1 binds to EZH2 and suppresses the enrichment of EZH2 on the c-Myc promoter, leading to the epigenetic up-regulation of c-Myc.

Unlike mRNAs, lncRNAs exhibit diverse subcellular distribution patterns, and their functions are closely associated with their specific localization.^5^^,^^41^ Several lncRNAs, including MALAT1, H19, MDRL, HBL1, LncMyoD, and PYCARD-AS1, localize to both cytoplasm and nucleus, where they regulate diverse cellular functions through distinct mechanisms.^42^, ^43^, ^44^ For example, nuclear PYCARD-AS1 interacts with DNMT1 and G9a to epigenetically regulate the transcription of PYCARD, while in the cytoplasm, PYCARD-AS1 suppresses PYCARD translation by blocking ribosome assembly.^43^ In this study, we observed that USP30-AS1 is localized in both cytoplasm and nucleus. In the cytoplasm, USP30-AS1 interacts with HnRNPF to prevent the binding between HnRNPF and p21 mRNA, resulting in the destabilization of p21 mRNA. In the nucleus, USP30-AS1 binds to EZH2, and suppresses the binding of EZH2 to the c-Myc promoter, leading to epigenetic up-regulation of c-Myc (Fig. 10). Collectively, this study reveals the coordinated regulation of target gene expression by USP30-AS1 in both nucleus and cytoplasm, highlighting its dual role in breast cancer progression.

## CRediT authorship contribution statement

**Yapei Jiang:** Writing – review & editing, Writing – original draft, Visualization, Validation, Supervision. **Weijie Liao:** Writing – review & editing, Validation, Conceptualization, Funding acquisition, Methodology. **Qilei Xin:** Validation. **Ruonan Wang:** Visualization. **Guanglan Lin:** Validation. **Jia Li:** Validation. **Zijian Yang:** Validation. **Shiyue Yang:** Validation. **Haowei Zhang:** Validation. **Xiaolin Li:** Validation. **Qian Peng:** Validation. **Yaou Zhang:** Writing – review & editing, Writing – original draft, Funding acquisition. **Weidong Xie:** Writing – review & editing, Funding acquisition, Conceptualization. **Naihan Xu:** Writing – review & editing, Writing – original draft, Funding acquisition, Formal analysis, Conceptualization.

## Data availability

All the data are available in the article and Supplementary files, or available from the authors upon request.

## Funding

This work was supported the 10.13039/501100012166National Key R&D Program of China (No. 2023YFA0914300, 2023YFA0914302), the 10.13039/501100001809National Natural Science Foundation of China (No. 31571418, 32200438), the 10.13039/501100010877Shenzhen Science and Technology Innovation Commission (No. JCYJ20160226185623304, JCYJ20240813142003005), and 10.13039/501100017700Henan Provincial Science and Technology Research Project (No. 252102310403).

## Conflict of interest

The authors declared no conflict of interests.
