# Supplementary material for: Nuclear and cytoplasmic USP30-AS1 coordinately regulate breast cancer progression through HnRNPF/p21 and EZH2/c-Myc/p21 axes
Source: Genes Dis. 2025 May 10;13(2):101684. doi: 10.1016/j.gendis.2025.101684 (PMC12765101; doi:10.1016/j.gendis.2025.101684)
Supplement: Multimedia component 1 [file mmc1.docx]

**Supplementary information**

**1. Materials and Methods**

**Transcriptome and Bioinformatics Analysis**

The expression signatures of USP30-AS1 were investigated in various subtypes of breast cancer samples from the TCGA dataset (https://portal.gdc.cancer.gov/) and the Cancer Cell Line Encyclopedia (CCLE) datasets (https://portals./ccle). We also download the GSE61304 microarray data from the GEO database (https://www.ncbi.nlm.nih.gov/geo/) to analyze the expression of USP30-AS1 in breast cancer and normal breast tissues.

Gene Set Enrichment Analysis (GSEA) can enrich signaling pathways by analyzing whole-genome expression profile chip data. We divided the gene expression profile of breast cancer into USP30-AS1 low expression and USP30-AS1 high expression groups based on the median expression of USP30-AS1, for functional enrichment and prediction of signaling pathways associated with USP30-AS1 expression. Signaling pathways with |ENS| > 1, FDR < 0.25, and p < 0.05 were considered significantly enriched. A higher value of |ENS| > 1 indicated a higher degree of enrichment.

The proteins to which USP30-AS1 is likely to bind can be predicted by CatRAPID omics v2.1 (http://s.tartaglialab.com/update_submission/616977/23c65a510d). CatRAPID fragment (http://s.tartaglialab.com/update_submission/616975/7f895cf456) is used to predict the specific binding sites of USP30-AS1 and HnRNPF, USP30-AS1 and EZH2, and P21 mRNA and HnRNPF.

We utilize the RNA-Protein Interaction Prediction (RPISeq) tool, available at http://pridb.gdcb.iastate.edu/RPISeq/index.html, to forecast the probability of lncRNA binding to proteins.

The potential upstream transcriptional regulators of USP30-AS1 were predicted using GeneCards website (http://www.genecards.org), USCC-JASPAR website (https://genome.ucsc.edu/index.html), and Animal TFDB website (https://guolab.wchscu.cn/AnimalTFDB/#/).

The correlation between the expression levels of transcription factors and USP30-AS1 in breast cancer tissues, triple-negative breast cancer (TNBC), and normal breast tissues were analyzed by analyzing the TCGA dataset. Potential SPI1 binding sites within the promoter region of USP30-AS1 were predicted using the Jaspar database (https://jaspar.genereg.net/).

**2. Supplementary Figures**


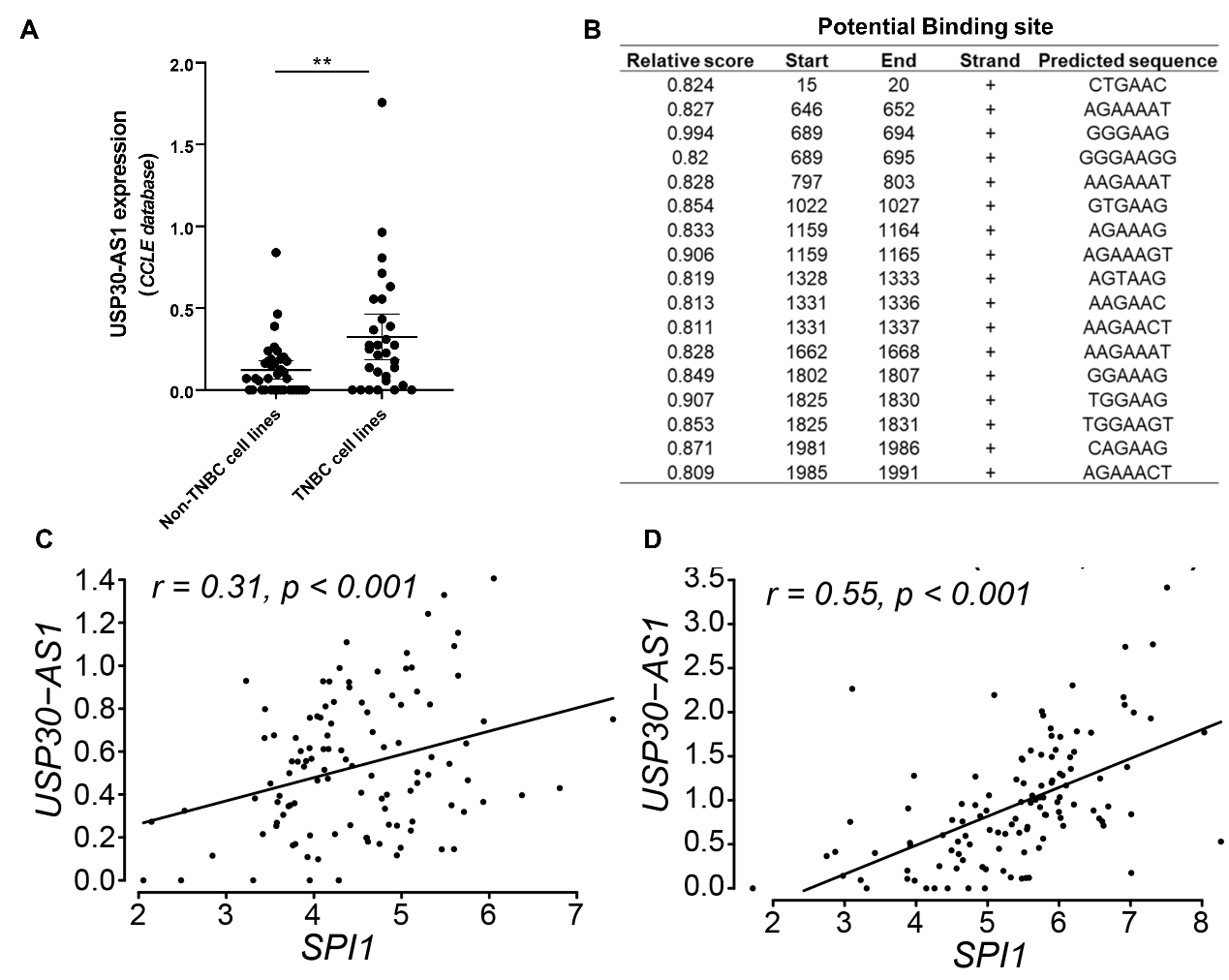


**Supplementary Figure 1:**

**(A).** The expression of USP30-AS1 in non-TNBC cell lines (n = 22) and TNBC cell lines (n = 24), data were obtained from CCLE datasets. **(B)** The potential binding sites between SPI1 and the promoter region of USP30-AS1 are analyzed by JASPAR web. The correlation between transcription factors SPI1 and USP30-AS1 **(C)** in normal breast tissues (n = 112) and **(D)** in TNBC tumors (n = 116) by analyzing TCGA datasets.


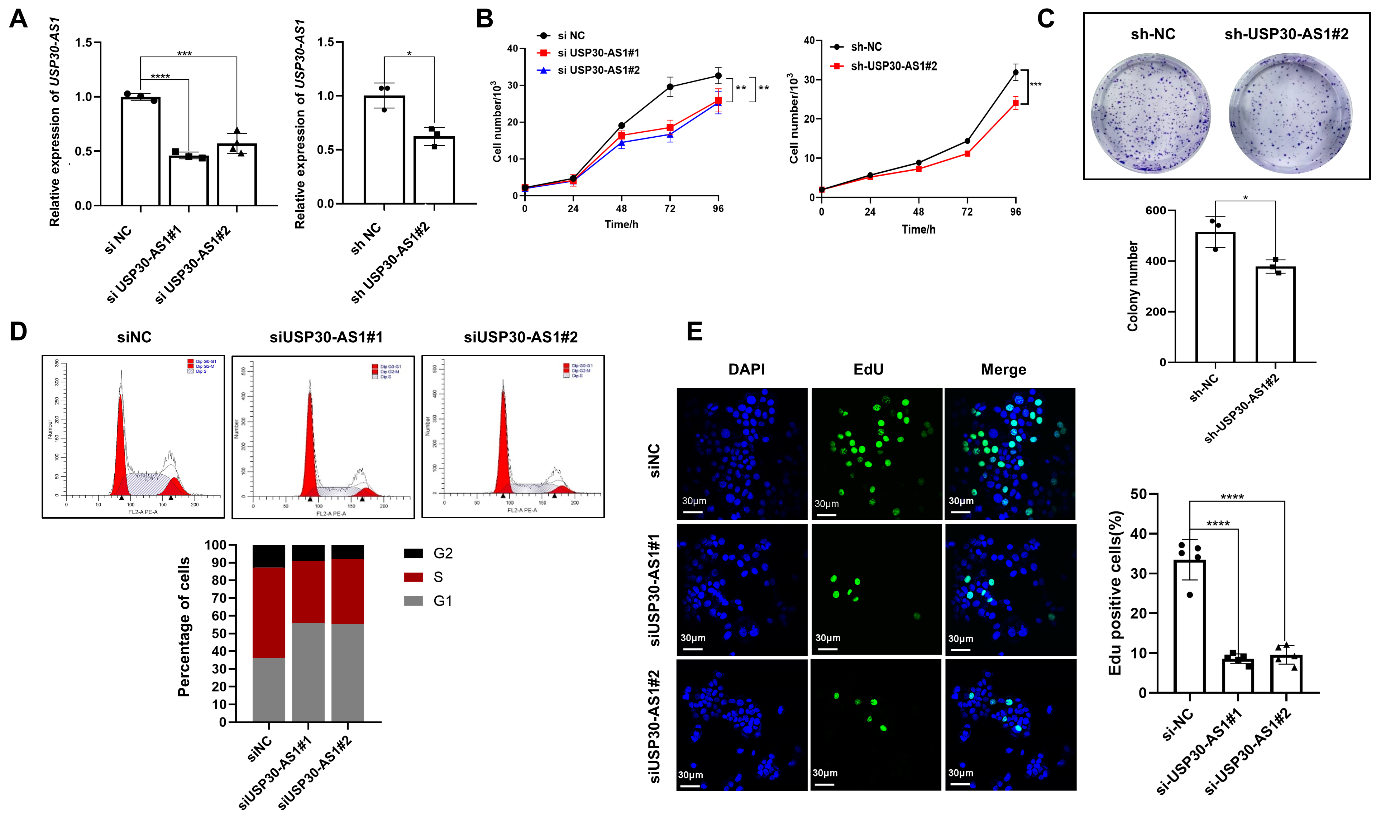
**Supplementary Figure 2:**

**(A)** The knockdown efficiency of USP30-AS1 on MCF-7 cell was verified *via* qRT-PCR assays. **(B)** The effect of USP30-AS1 knockdown on MCF-7 cell proliferation was evaluated by CCK-8 assays. **(C)** The impact of USP30-AS1 knockdown on the proliferative capacity of MCF-7 cell was assessed by colony formation assays. **(D)** Flow cytometry analysis and statistical analysis were conducted to determine effect of USP30-AS1 knockdown on the cell cycle progression of MCF-7 cells. **(E, F)** EdU assays were performed in MCF-7 cells with USP30-AS1 knockdown.


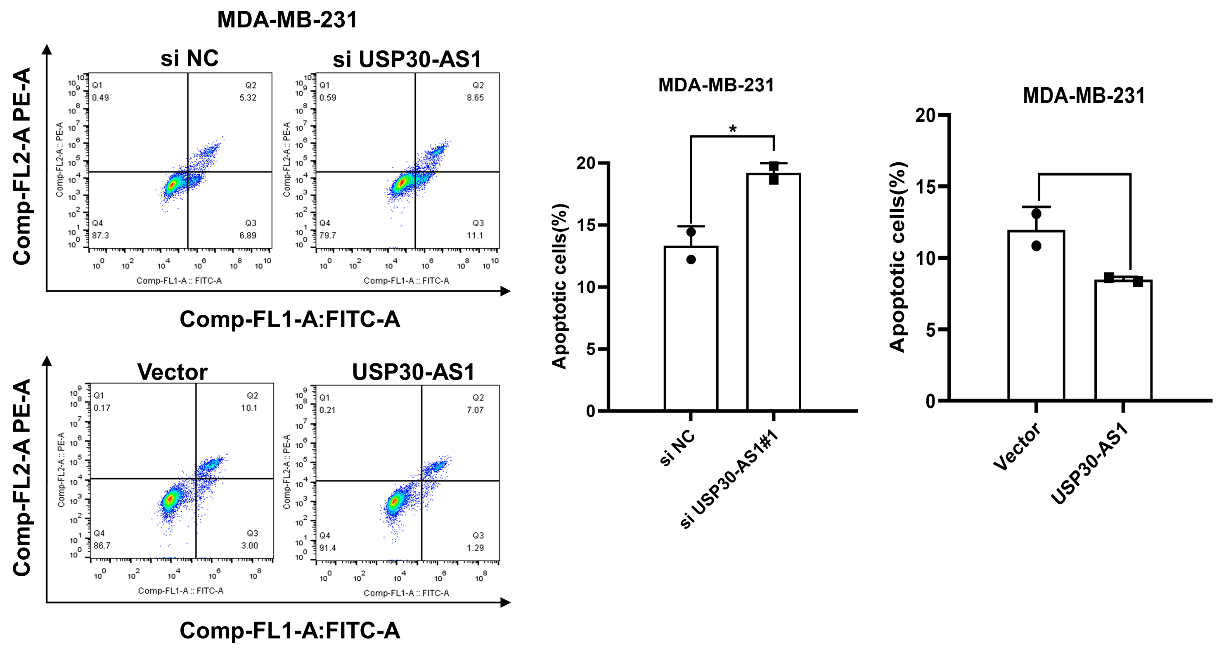


**Supplementary Figure 3:**

Annexin V/PI flow cytometry analysis and subsequent statistical analysis were conducted to determine the role of USP30-AS1 on apoptosis in MDA-MB-231 cells.


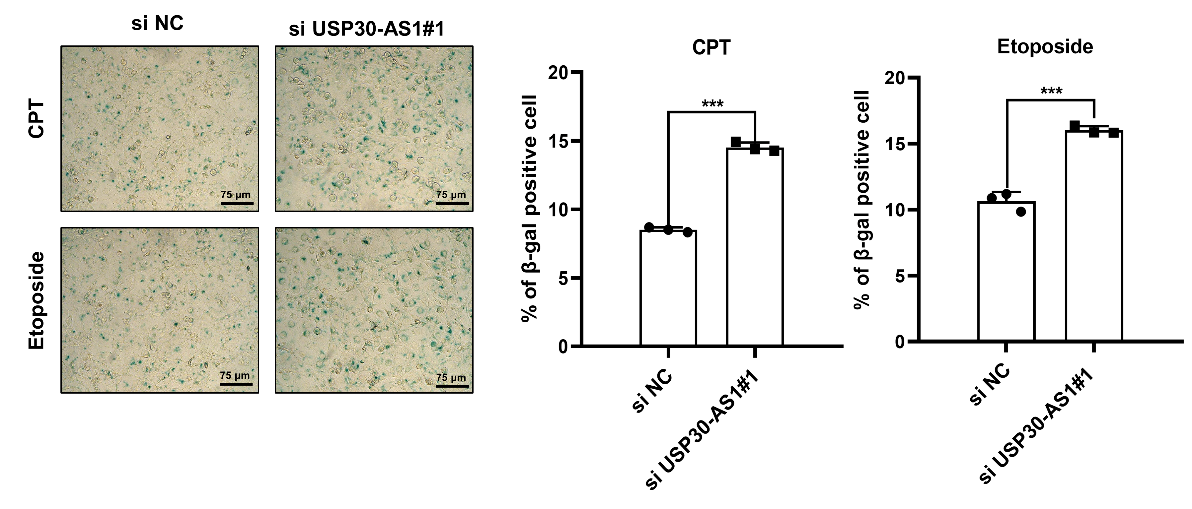


**Supplementary Figure 4:**

After transfecting MDA-MB-231 cells with siRNA to knockdown USP30-AS1, treatments were applied with 50 nM Camptothecin or 10 μM Etoposide to induce for 48 hours. Cellular senescence was assessed through β-galactosidase assays to detect β-galactosidase-positive cells. The left panel displays the staining results, while the right panel presents the statistical analysis of the proportion of positive cells.


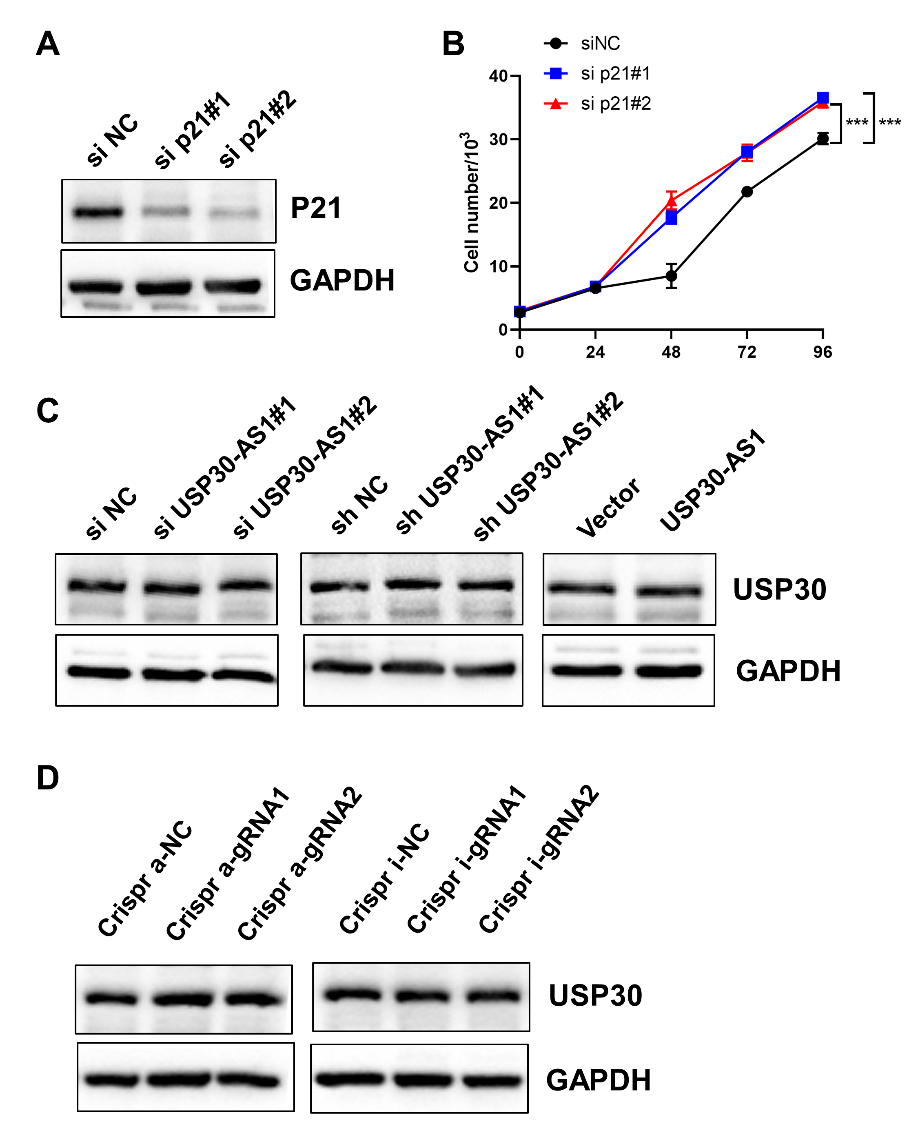


**Supplementary Figure 5:**

**(A)** In MDA-MB-231 cells, siRNA was employed to knock down p21 expression, and Western blot analysis was used to assess the expression of p21 protein. **(B)** The impact of p21 knockdown on the proliferation of MDA-MB-231 cells was assessed by using the CCK-8 assay. **(C)** In MDA-MB-231 cells, after knocking down USP30-AS1 using siRNA and shRNA or overexpressing USP30-AS1 by transfecting exogenous plasmids, the effect of USP30-AS1 on USP30 was validated by Western blot. **(D)** In HEK293 cells, after knocking down and overexpressing USP30-AS1 using CRISPRi and CRISPRa respectively, the effect of USP30-AS1 on USP30 was validated by Western blot.


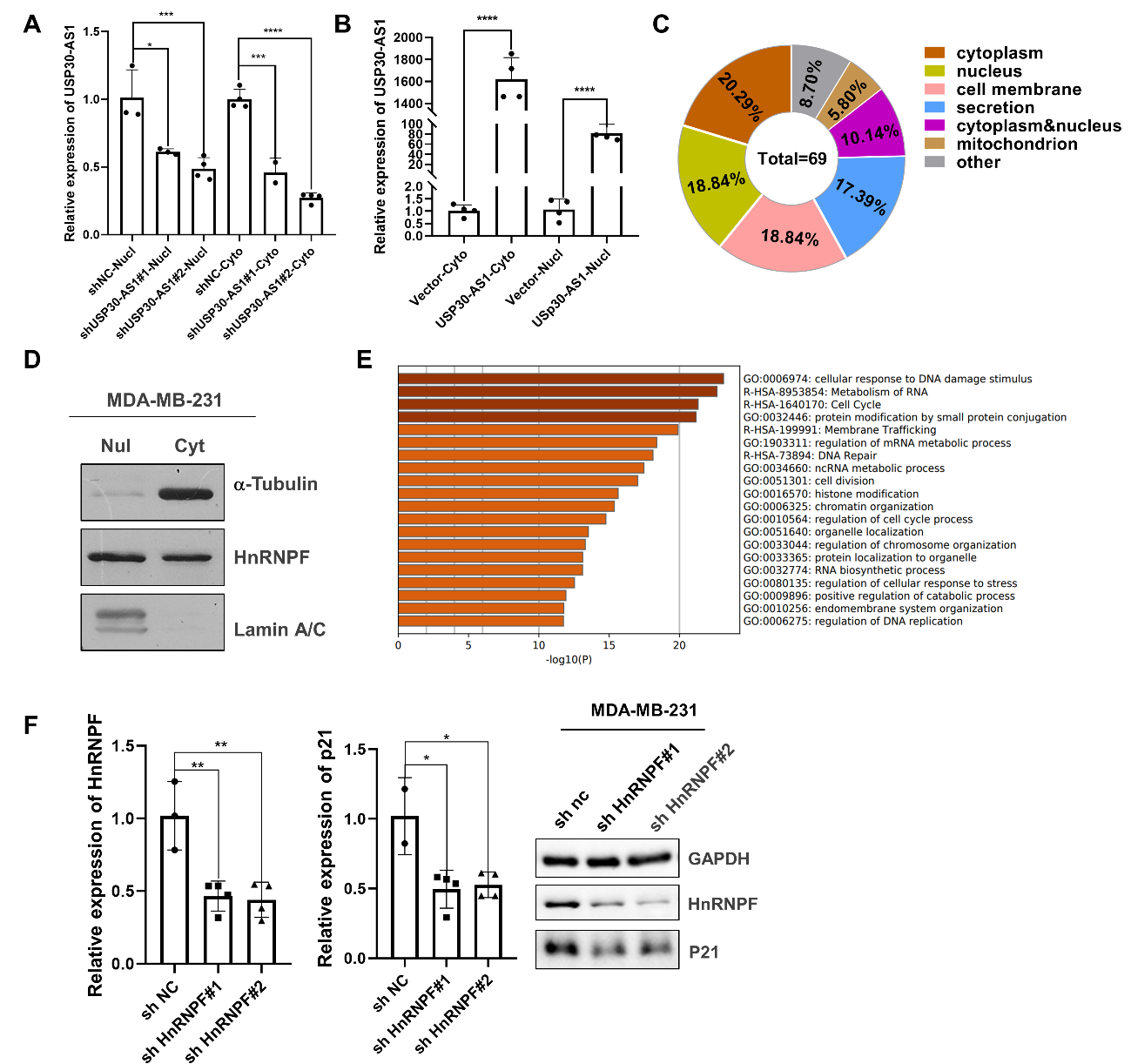


**Supplementary Figure 6:**

**(A)** In MDA-MB-231 cells, USP30-AS1 knockdown was performed, and nuclear-cytoplasmic fractionation followed by qRT-PCR analysis confirmed a reduction in USP30-AS1 expression in both the nucleus and the cytoplasm. **(B)** In MDA-MB-231 cells overexpressing USP30-AS1, nuclear-cytoplasmic fractionation followed by qRT-PCR analysis confirmed increased USP30-AS1 expression in both the nucleus and the cytoplasm. **(C)** The subcellular localization of the specific proteins identified by RNA pull down that bind USP30-AS1. **(D)** Nuclear-cytoplasmic fractionation was executed in MDA-MB-231. The HnRNPF expression in the nucleus and cytoplasm was carried out through western blot assay, employing Lamin A/C and α-Tubulin as distinguishing markers for the nucleus and cytoplasm, respectively. **(E)** Metascape illustrates the top enriched clusters and their enrichment patterns across several gene lists (HnRNPF-correlated genes in breast cancer, data from GEPIA. **(F)** HnRNPF was knocked down by shRNA in MDA-MB-231 cells, and the mRNA and protein expression of HnRNPF and P21 were analyzed by qRT-PCR and western blot, respectively.


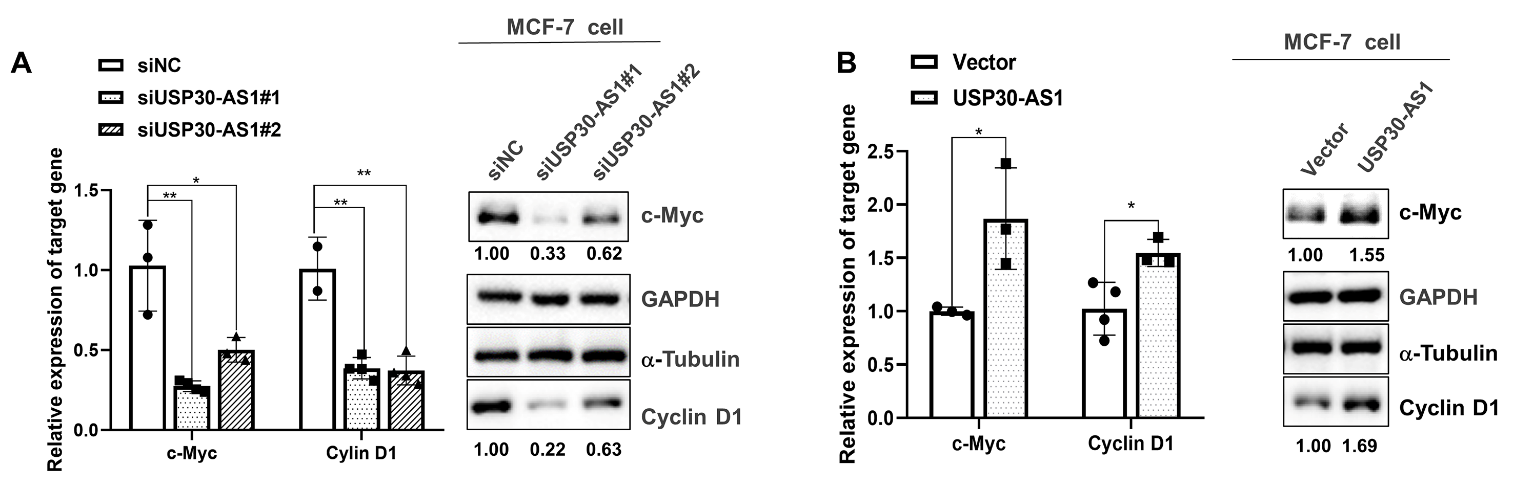


**Supplementary Figure 7:**

**(A)** Following the knockdown of USP30-AS1 by siRNA in MCF-7 cell, the expressions of c-Myc and Cyclin D1 at both the mRNA and protein levels were evaluated using qRT-PCR and western blot, respectively. **(B)** After overexpression of USP30-AS1 through transfection of exogenous plasmid in MCF-7 cell, the expression levels of c-Myc and Cyclin D1 were assessed at both the mRNA and protein levels using qRT-PCR and western blot, respectively.


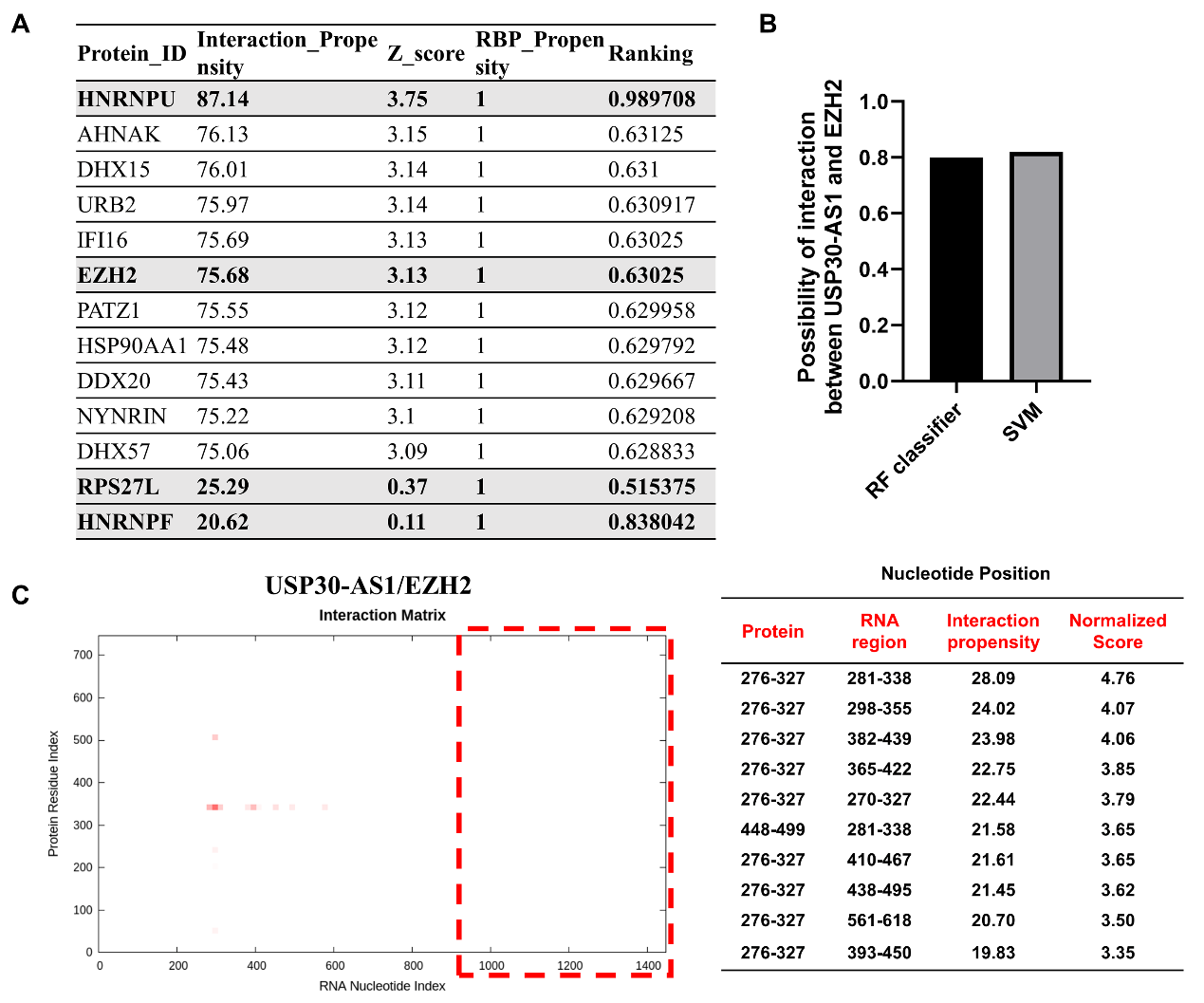


**Supplementary Figure 8: The prediction between USP30-AS1 and EZH2**

**(A)** The catRAPID website was utilized to predict proteins potentially interacting with USP30-AS1. **(B)** Applying the algorithmic support provided by the RPISeq database to analyze the potential interaction between USP30-AS1 and EZH2.
